# Magnetic-guided nanocarriers for ionizing/non-ionizing radiation synergistic treatment against triple-negative breast cancer

**DOI:** 10.1186/s12938-024-01263-7

**Published:** 2024-07-13

**Authors:** Yun Zhou, Junhao Kou, Yuhuang Zhang, Rongze Ma, Yao Wang, Junfeng Zhang, Chunhong Zhang, Wenhua Zhan, Ke Li, Xueping Li

**Affiliations:** 1 College of Clinical Medicine, Xi’an Medical University, Xi’an, 710021 China; 2 Xi’an Key Laboratory for Prevention and Treatment of Common Aging Diseases, Translational and Research Centre for Prevention and Therapy of Chronic Disease, Institute of Basic and Translational Medicine, Xi’an Medical University, Xi’an, 710021 China; 3 College of Pharmacy, Xi’an Medical University, Xi’an, 710021 China; 4 Department of Radiation Oncology, First Affiliated Hospital of Xi’an Jiaotong University, Xi’an, 710061 China; 5 Xi’an Key Laboratory of Advanced Control and Intelligent Process, School of Automation, Xi’an University of Posts & Telecommunications, Xi’an, 710121 China; 6 Department of Radiotherapy, General Hospital of Ningxia Medical University, Yinchuan, 750004 China

**Keywords:** Triple-negative breast cancer, Radiotherapy, Ionizing radiation, Non-ionizing radiation, Synergistic therapy

## Abstract

**Background:**

Triple-negative breast cancer (TNBC) is a subtype of breast cancer with the worst prognosis. Radiotherapy (RT) is one of the core modalities for the disease; however, the ionizing radiation of RT has severe side effects. The consistent development direction of RT is to achieve better therapeutic effect with lower radiation dose. Studies have demonstrated that synergistic effects can be achieved by combining RT with non-ionizing radiation therapies such as light and magnetic therapy, thereby achieving the goal of dose reduction and efficacy enhancement.

**Methods:**

In this study, we applied FeCo NPs with magneto thermal function and phototherapeutic agent IR-780 to construct an ionizing and non-ionizing radiation synergistic nanoparticle (INS NPs). INS NPs are first subjected to morphology, size, colloidal stability, loading capacity, and photothermal conversion tests. Subsequently, the cell inhibitory and cellular internalization were evaluated using cell lines in vitro. Following comprehensive assessment of the NPs’ in vivo biocompatibility, tumor-bearing mouse model was established to evaluate their distribution, targeted delivery, and anti-tumor effects in vivo.

**Results:**

INS NPs have a saturation magnetization exceeding 72 emu/g, a hydrodynamic particle size of approximately 40 nm, a negatively charged surface, and good colloidal stability and encapsulation properties. INS NPs maintain the spectral characteristics of IR-780 at 808 nm. Under laser irradiation, the maximum temperature was 92 °C, INS NPs also achieved the effective heat temperature in vivo. Both in vivo and in vitro tests have proven that INS NPs have good biocompatibility. INS NPs remained effective for more than a week after one injection in vivo, and can also be guided and accumulated in tumors through permanent magnets. Later, the results exhibited that under low-dose RT and laser irradiation, the combined intervention group showed significant synergetic effects, and the ROS production rate was much higher than that of the RT and phototherapy-treated groups. In the mice model, 60% of the tumors were completely eradicated.

**Conclusions:**

INS NPs effectively overcome many shortcomings of RT for TNBC and provide experimental basis for the development of novel clinical treatment methods for TNBC.

**Supplementary Information:**

The online version contains supplementary material available at 10.1186/s12938-024-01263-7.

## Background

Triple-negative breast cancer (TNBC) is a special subtype of breast cancer. It does not express estrogen receptor (ER), progesterone receptor (PR), and human epidermal growth factor receptor 2 (HER2), accounting for 10–20% of the breast cancer cases [1–3]. TNBC has the characteristics of early onset, late clinical stage, high histological grade, strong invasiveness, and high heterogeneity. Due to the lack of effective targets on TNBC cells, the treatment methods do not meet the expectation, and the prognosis is very poor. The median survival duration of advanced TNBC is only about 1 year [4, 5]. For highly malignant tumors of TNBC, the effect of a single treatment method is very limited. Using a combination of existing treatments to improve the efficacy of TNBC is the focus of current TNBC therapy research [6]. Radiotherapy (RT) is an important part of the TNBC treatment and is crucial to the long-term survival and life quality of patients [7, 8]. However, as a treatment method based on high-energy ionizing radiation, RT has its shortcomings. Firstly, ionizing radiation will damage surrounding normal tissues while killing tumor cells. As a soft tissue, tumor has a weak interaction with ion beams, and a large amount of radiation energy will be applied to the adjacent normal tissues, causing serious side effects. Secondly, the tumor microenvironment, including hypoxia, insufficient endogenous H_2_O_2_, and high concentrations of glutathione, will reduce the efficiency of RT [9, 10]. Combining with other methods to improve RT sensitivity, reduce the radiation dose, and comprehensively improve the treatment effect is an effective way to solve the problems of RT [11, 12].

In addition to ionizing radiation used in RT, radiation also includes non-ionizing radiation (NIR) with lower energy levels. NIR is divided into two main fields, optical radiation and electromagnetic fields. NIR is commonly used in healthcare applications including ultrasound imaging, laser surgery, and phototherapy (PT) [13, 14]. PT, as a minimally invasive NIR treatment method, has been widely explored in tumor treatment due to its unique advantages including simplicity, high efficiency, and low treatment resistance [15, 16]. PT relies on phototherapy agents (PAs) to convert light energy into chemical (photodynamic therapy, PDT) or thermal energy (photothermal therapy, PTT), thereby triggering a series of reactions in the body to destroy target cells, while significantly reduces the side effects on normal tissues [17, 18]. The combination of PT and RT is also very compatible. Combining with RT, PTT can directly kill tumor cells by generating heat, which includes a large number of S-phase and hypoxic area cells within tumor that are insensitive to RT. PTT can also effectively prevent post-RT recovery of damaged cell. Additionally, PTT can accelerate blood flow within the tumor, relieve tumor hypoxia, and improve RT sensitivity, thereby further improving the effect of RT [19–21]. Xiao et al. developed a core–shell structure nanoparticle with CuS and silicon materials coated rare earth core, achieving in vivo imaging and a synergy between efficient RT and PTT [22]. Zhang et al. used iRGD peptides to modify Fe and Pt loaded Au nanocages to prepare a multifunctional nanocarrier for breast cancer. While achieving synergy effect by inducing ferroptosis, the nanocarrier could also monitor the therapeutic effect with imaging [23]. Peng et al. constructed an oxygen-carrying photothermal nanocarrier containing Ta, which released oxygen through near-infrared laser excitation. At the same time, photothermal promoted blood flow to further alleviate hypoxia and caused direct killing of tumor cells. The Ta element combined with RT also caused multiple effects [24]. Yong et al. designed a nanocarrier combined with radio-sensitization and PTT, which can also achieve X-ray and photoacoustic dual-modal imaging. The nanoparticles were delivered through intravenous injection and could effectively accumulate in the tumor site to achieve joint tumor inhibition [25]. Other than PTT, there are also many studies on combining RT and PDT, such as X-ray–PDT, proton-induced PDT, etc. This new field is called radio-dynamic therapy. It has the potential to achieve higher synergy in tumor treatment, maximizing the dosage to the diseased site while avoiding damage to healthy tissue [26]. Bulin et al. found that low-dose PDT and RT worked synergistically to produce beneficial effects on pancreatic heterogeneous spheroids [27]. Wang et al. used radionuclides as the internal excitation sources to activate photosensitizers for PDT of deep tumors, achieving an effective combination of RT and PDT and showing excellent tumor inhibition ability [28]. Dan et al. used ICG and ultra-small Au NCs to construct a nanozyme system that integrated treatment and diagnosis to regulate tumor hypoxia and enhance the effects of cancer PDT and RT, while taking advantage of the X-ray absorption of Au NCs. The ability greatly improved the radiation energy deposition within the tumor area and further improved cancer RT [29].

Many studies have demonstrated that nanomaterials can be utilized as sensitizers or enhancers for RT, significantly enhancing its effectiveness on tumor areas, minimizing radiation dosage, and mitigating side effects. For example, nanoparticles with high atomic numbers prone to absorb radiations. When they are accumulated into tumor tissue and irradiated with high-energy radiation, the particles will exhibit a variety of effects after absorbing the radiation, releasing photoelectrons, Compton electrons, Auger electrons, and that can react with organic molecules or water in tumor cells to generate a large number of free radicals, which can enhance the effect of RT [30]. Moreover, nanomaterials can not only promote the damage effect of RT through interaction, but also load various compounds to achieve synergistic treatment of chemotherapy, hyperthermia, PDT and RT, overcoming the shortcomings of single RT and achieving complementary advantages and synergistic effects. Ferrite nanomaterial is an effective RT sensitizer, which catalyzes the generation of free radicals with killing effects and achieves RT sensitization effects. Studies have shown that Fe_3_O_4_, especially superparamagnetic Fe_3_O_4_ nanoparticles (SPIONS), has a RT dose-enhancing effect [31]. Co and Fe both are Group VIII elements. It has been reported that Co and Fe can form a spinel structure and be used in high-temperature treatment with remarkable therapeutic results [32, 33]. The FeCo NPs prepared by Zhang et al. were used for magnetic hyperthermia therapy of tumors, showing clear therapeutic advantages [34]. However, the surface of FeCo NPs is hydrophobic and requires surface modification before applying in vivo. There are many reports on surface modification processes. However, the required materials need to meet biocompatibility requirements and cover and encapsulate other functional molecules easily and reliably. Polydopamine (PDA) is a biocompatible biopolymer which mainly formed by the rapid oxidative self-polymerization of dopamine through hydrogen bonds, covalent bonds, π–π interactions, etc. Lee et al. created an adhesive multifunctional PDA coating on the surface of various types of organic and inorganic materials. Since then, PDA has received widespread attention as a new material [35]. Coating PDA and functional substances on the surface of nanoparticles to prepare nanocomposites is an effective method to improve the dispersion of nanoparticles. PDA is an ideal material for this study since it can effectively carry various active molecules through π–π stacking, hydrogen bonding, and van der Waals interactions [36, 37].

**Fig. 1:**
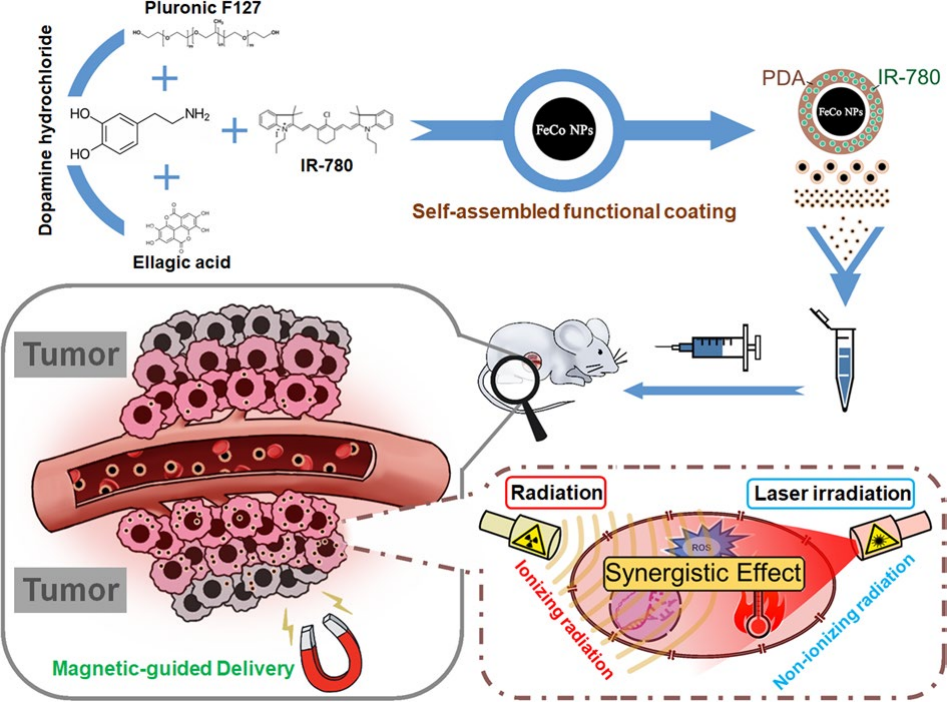
Scheme of INS NPs’ functional design

In this study, based on the FeCo NPs prepared by Zhang et al. [34], we applied PDA to load phototherapy agent IR-780 on the surface of FeCo NPs to obtain a multifunctional nanoparticle, INS NPs. The particle size and surface properties of INS NPs ensure their good circulation and tumor accumulation effects in the body. The strong magnetism due to the FeCo core enabled tumor targeted delivery through magnetic guidance and retained the potential of magnetothermal and MRI T2-weighted imaging. IR-780 is a phototherapy molecule with both PTT and PDT capabilities, and can be combined with FeCo NPs to enhance the effect of RT. The INS NPs achieved a synergistic therapeutic effect of ionizing radiation and non-ionizing radiation, and provides a basis for the development of new clinical treatment methods for TNBC. The delivery and combined intervention of INS NPs are illustrated in Fig. 1.

## Materials and methods

### Materials

Pluronic F127, ellagic acid, IR-780, dopamine hydrochloride, coumarin-6, and doxorubicin (DOX) were purchased from Shanghai Macklin Ltd. DMEM high-glucose medium, RPMI 1640 medium, and trypsin were purchased from Cytiva Hyclone PLC. Cell culture antibiotics, CCK-8, DAPI kit, paraformaldehyde solution, TUNEL stain, HE staining, and ROS kit were purchased from Shanghai Beyotime Ltd. Dihydroethidium (DHE) ROS fluorescent probe was purchased from Beijing Solarbio Ltd. All the other reagents were purchased from Sinopharm Corp. FeCo NPs were synthesized in our lab based on the procedures in Zhang's publication. The 3T3-L1 (mouse embryonic fibroblast cell line), HUVEC (human umbilical vein endothelial cell lines), IMR-90 (human lung diploid fibroblast cell line), HL-7702 (human liver cell line), MCF-10a (non-tumorigenic human mammary epithelial cell line), and 4T1 (mouse mammary carcinoma cell line) cell lines were derived from ATCC. All experimental animals were purchased from Beijing HFKBio PLC.

### Synthesis of INS NPs

36 mg of Pluronic F127, 1 mg of ellagic acid, and 12 mg of IR-780 were weighed and dissolved in 1 mL DMSO. After fully dissolved, the mixed solution was added dropwise to 8 mL of a Tris solution containing 10 mg of FeCo NPs (pH = 9.0), ultrasonicated for 10 min, and added with 60 mg of dopamine hydrochloride. The mixed solution was sealed and rotated for polymerization for 48 h. The solution was then dialyzed with Tris solution for 24 h, during which the liquid was replaced until the external liquid was clear. The prepared dispersion was stored overnight. After observing whether there was precipitation, we measured its hydrodynamic size and zeta potential distribution with a Malvern particle size analyzer (Nano ZS, Malvern, UK).

### Characterization of INS NPs

The morphology of FeCo NPs and INS NPs was observed using transmission electron microscopy (TEM, Tecnai G2 F20, FEI, US). Both NPs were dispersed in PBS, complete culture medium, and serum, respectively. The changes in particle size distribution were continually measured for colloidal stability. The nanoparticles were dispersed in PBS, complete culture medium, and serum, stored at 4 °C, and observed for particle size changes within 15 days. After the NPs were freeze-dried, they were soaked in acidic DMSO to extract IR-780, and the encapsulation rate and drug loading capacity were measured. The concentration of IR-780 in the sample after 72 h was measured by dialysis method to determine the release brief. After freeze-drying and grinding the NPs, we tested the magnetism of the sample using a permanent magnet. The magnetism was tested using a vibrating sample magnetometer (Lakeshore, US). The absorption spectra of the samples were measured using a UV–visible spectrophotometer (T6, Persee, Shanghai, China). The fluorescence emission spectrum of the sample was measured using a fluorescence spectrophotometer (F98, SAID, Shanghai, China). The T2 relaxation rate of the samples was measured using a small animal MRI system (NM21-060H-I, NIUMAG, Suzhou, China). In terms of photothermal conversion, an 808 nm laser (BOT808-5W-GQ, BOT, Xi’an, China) was used to irradiate the sample under different conditions, and an infrared thermal imaging camera (EX6, FILR, US) was used to monitor its temperature changes. Except for the explicitly marked tests, the concentration of INS NPs used for all tests was 200 μg/mL. The concentration of the component was designed based on the preliminary calculation of the drug loading. Then, adipose and muscle tissue were covered on the surface of the sample solution for in vitro heating testing under block. The BALB/c mice were used to test the subcutaneous and intramuscular heating effects of the sample in vivo.

### Cellular internalization and affinity test of INS NPs

The green fluorescent dye coumarin-6 and the red fluorescent molecule DOX were used to label INS NPs. 4T1 cells were cultured in a 3.5 cm confocal culture dish at a density of 20,000 cells/dish. After the cells were growing well, fluorescence-labeled INS NPs were added and we collected samples at different time points. After paraformaldehyde fixation, the nuclei were stained with DAPI, and the cellular internalization was observed by confocal microscopy (FV3000, Olympus, Japan). The 4T1 cells were pretreated with the active endocytosis inhibitor, NaN_3_ (concentration 1 mg/mL) for 1 h, and then fluorescence-labeled INS NPs were added to the cells. After incubation for 8 h, the cells were fixed with paraformaldehyde, and the nuclei were stained with DAPI. Intracellular fluorescence levels were detected and quantified by laser confocal microscopy.

### In vitro anti-tumor test of INS NPs

The cytotoxicity of INS NPs and main components was tested by CCK-8 assay. The normal cell lines (3T3-L1, HUVEC, IMR-90, HL-7702, MCF-10a) and TNBC cell line 4T1 were planted in 96-well plates at 8000–12,000 cells/well. INS NPs and the components in concentration gradients were added to the wells. There were three duplicated wells for each concentration. The cells were cultured at 37 °C with 5% CO_2_. When the cells in control wells were reaching a density of 90%, we replaced the medium with colorless medium containing 10% of CCK-8. The plates were incubated for 1–1.5 h before reading at 450 nm with a microplate reader (Spark, TACAN, US) to calculate the cell survival rate. The cell intervention effect of INS NPs was tested through the colony formation method. In a 6-well plate, 4T1 cells were seeded at 2000 cells/well. After the cells adhered, INS NPs samples were added and co-incubated for several hours, and PTT (1W/cm^2^, 3 min) and RT (2 Gy) were performed (Versa HD, Elekta, Beijing, China). Only PPT and only RT-treated wells were set as positive controls. Then the cells are cultured until the cell number in most colonies in the negative control wells was greater than 50. We then stained the cells and the number of colonies formed was calculated. In the live and dead cell staining test, 40,000 cells were inoculated in each well of a 6-well plate. After the cells grew to 70% density, intervention was carried out in the same grouping and conditions as for colony formation. The cells were then incubated for 12 h and stained with live and dead cells staining kit. The results were observed with a fluorescence microscope (DMi8, Leica, Germany). To test ROS in RT, 4T1 cells are inoculated in a 6-well plate at 40,000 cells/well. After adhered and grew well, the cells were divided into groups, given corresponding samples, and intervention. After incubating for 6 h we used the ROS kit to stain the cells and observed the fluorescence signals in the cells through a fluorescence microscope and take pictures. We use ImageJ to quantitatively analyze the fluorescence intensity in the cells. The ROS-enhancing ability of INS NPs was then further quantitatively analyzed by flow cytometry (FACSCanto II, BD, US).

### Biocompatibility evaluations of INS NPs

As intravenously administered, INS NPs need to be tested with hemolysis. We dispersed INS NPs and its main components in 2% red blood cell suspension, incubated at 37 °C for 2 h, centrifuged at 8000 rpm for 10 min, and observed the precipitation. Since the sample was too dark to measure the absorbance, a second hemolysis will be performed. We discarded the supernatant, added 1 mL water, incubated at 37 °C for 3 h, centrifuge at 8000 rpm for 10 min, measured the absorbance of the supernatant at 540 nm, and calculated the hemolysis rate.

An acute toxicity experiment was performed. Forty 8-week-old BALB/c mice, half male and half female, were placed in SPF room for one week to adapt. Saline, INS NPS, IR-780, and FeCo NPs were then intravenously injected into each group of mice. The sample concentration was the highest concentration to maintain their stability in the preparation, and the injection volume was 200 μL. After the injection, the adverse symptom, death, and weight changes of the mice were observed for 14 days. Pathological, biochemical, and inflammatory tests were carried out on 24 8-week-old female BALB/c mice, which were also placed in SPF lab for a week to adapt. After the mice were euthanized, we randomly selected mice from each group to collect their heart, liver, spleen, lung, and kidney to perform HE staining and to observe the pathological changes. We fully respect animal welfare. All animal experiments were conducted in accordance with the experimental methods approved by the Ethics Committee of Xi’an Medical University (approval document number: XYLS-2022188).

### Construction of tumor-bearing mice model

We selected 6-week-old female BALB/c-nu/nu nude mice and placed them in a SPF lab for one week to adapt. The 4T1 cells cultured to the logarithmic phase were digested with trypsin and resuspended in serum-free DMEM medium (1 × 10^7^ cells/mL). We subcutaneously injected 100–150 μL of the cell suspension into the crotch of the mice. When the tumors grew to a suitable volume, the mice model could be used for the rest experiments.

### In vivo distribution and tumor magnetic guidance delivery test of INS NPs

The single-crotch tumor-bearing mice were selected and divided into two groups. INS NPs and the same concentration of IR-780 were intravenously injected, respectively. The mice were imaged through a small animal imaging system (Visque, in vivo Smart-LF, Korea) at different time points. We observed the distribution and performed quantitative analysis of fluorescence signals in the mice with the excitation of 780 nm and emission of 820 nm. After the fluorescence signal in vitro weakened, the mice were euthanized to obtain the main organs and tumor. The fluorescence distribution and signal intensity of organs and tumor were observed and quantitatively analyzed with the same parameters.

The double-crotch tumor-bearing mice were selected and divided into two groups. INS NPs and the same concentration of IR-780 were intravenously injected, respectively. The right tumor area of each mouse was magnetically guided with a permanent magnet. The distribution of fluorescence signals in mice was observed at different time points using a small animal imaging system. After the fluorescence signal in the tumor area reached the highest level, the mice were euthanized to obtain the tumors. The tumor fluorescence distribution and signal intensity were observed quantitatively analyzed with the same parameters.

### In vivo anti-tumor effect test of INS NPs

Thirty single-crotch tumor-bearing mice were selected and divided into 5 groups, including normal saline, RT, INS NPs + laser, INS NPs + RT, and INS NPs + laser + RT groups. The intravenous injection dose is 1/5–1/10 of the highest tolerated concentration of the acute toxicity experiment of INS NPs. The laser irradiation time point was determined based on the results of in vivo distribution and magnetic guidance delivery experiments. The laser parameters were 808 nm, 1 W /cm^2^, and 3 min. RT was performed 4–6 h after the first laser irradiation with a dose of 4 Gy. The laser irradiation was conducted twice a week thereafter. About 24 h after the first RT, the mice from each group were randomly selected to be euthanized to collect tumor. The tumor was then embedded in OCT gel, pre-freeze on dry ice for about 5 min, quickly frozen in liquid nitrogen. The tissue ROS level was detected by DHE kit. The tumors were observed for three weeks. The tumor volume was measured to draw a tumor growth curve. The weight changes of the mice in each group were recorded as well. After three weeks, the mice that still had tumors were euthanized, and the tumor were collected for observing and weighing. The remaining mice without an obvious tumor were observed for 14 days to record the tumor recurrence before being euthanized. The tumor tissues were collected, photographed, weighed, and embedded in paraffin. The sections were stained with HE and TUNEL for pathological analysis.

### Statistical analysis

All experiments were repeated at least three times independently. All data are expressed as mean value ± SD. The two-way ANOVA test was performed to determine if the difference was statistically significant or not (*p < 0.05, **p < 0.01, and ***p < 0.001).

## Results

### Performance of INS NPs

**Fig. 2:**
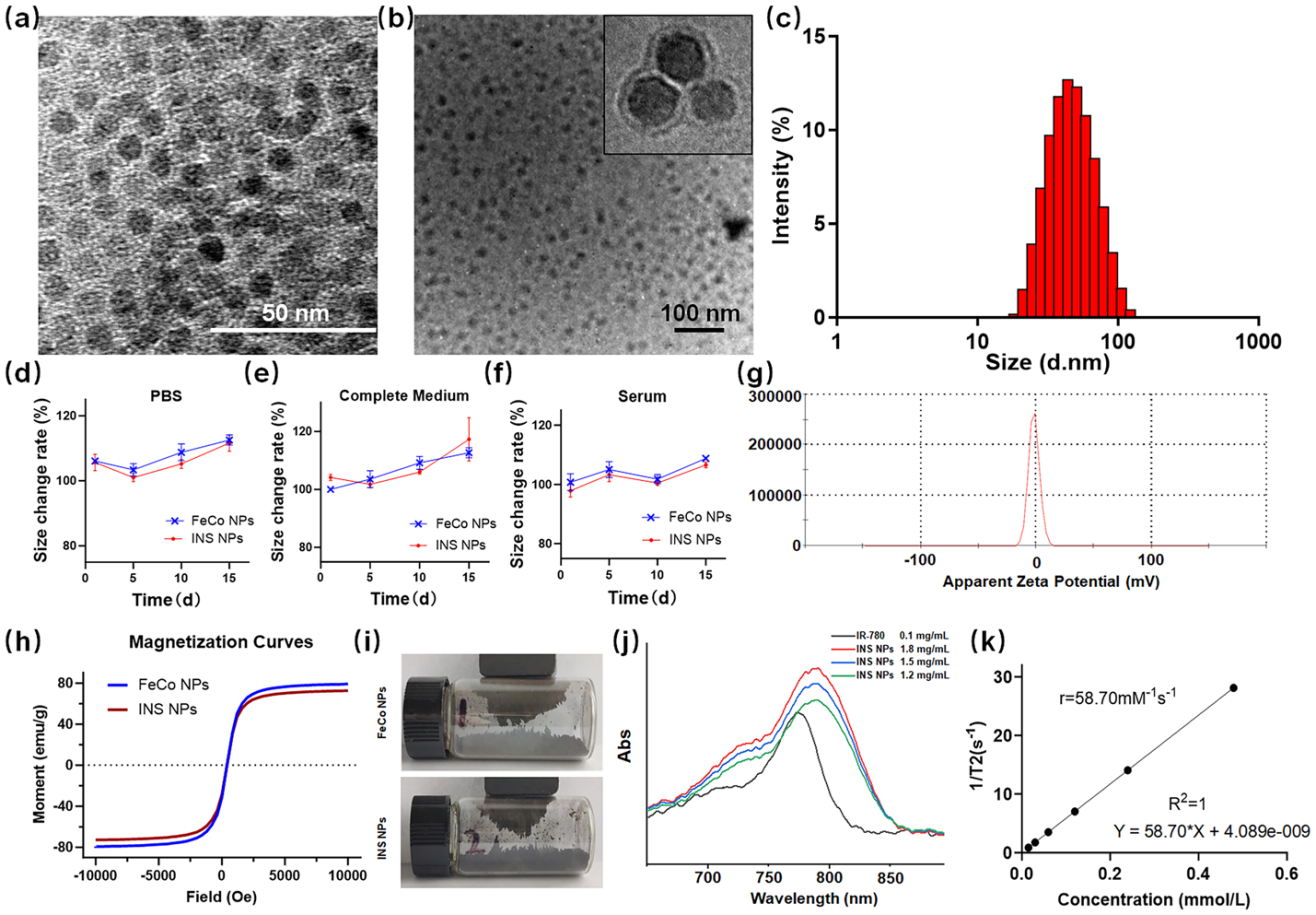
The main characteristic results of INS NPs: **a** TEM picture of FeCo NPs; **b** full view and detailed TEM pictures of INS NPs; **c** the size distribution of INS NPs; **d–f** particle size change curves (*n* = 3) of FeCo NPs and INS NPs in PBS, complete culture medium, and serum, respectively, The storage temperature of solution was 4 °C; **g** the zeta potential distribution of INS NPs; **h** the magnetization curves of FeCo NPs and INS NPs; **i** the magnetic attraction results of FeCo NPs and INS NPs; **j** the absorption spectrum of INS NPs and IR-780; **k** the relaxation rate measurement results of INS NPs

As shown in the TEM images (Fig. 2a–b), INS NPs had a PDA hybrid layer coated on the surface of FeCo NPs, which is consistent with the design. The size of INS NPs from TEM was approximately 20 nm, while the hydrodynamic size distribution was 41.21 ± 4.8 nm (Fig. 2c). The zeta potential of INS NPs in neutral pH solution is approximately −1 mV, which is essentially electrically neutral (Fig. 2g). The discrepancy of size is due to the hydration layer on the surface of INS NPs. This size is very conducive to the long circulation of NPs in the body [38]. It can be seen in Fig. 2g that the surface of INS NPs had a slightly negative charge, which also helps to reduce the chance of being cleared in the body circulation. The colloidal stability test results are shown in Fig. 2d-f. The particle size change rates of INS NPs in PBS, complete culture medium, and serum within 2 weeks were all within 10%, showing a good stability. INS NPs’ encapsulation rate for IR-780 was more than 92%, and the release rate within 72 h was very low (Fig. S1). With regard to the magnetization properties, the saturation magnetization intensity of FeCo NPs was approximately 79 emu/g, which was consistent with the literature report [34]. However, the saturation magnetization intensity of the INS NPs had an insignificantly decrease of 8%. It can be seen from Fig. 2h and i, through the permanent magnet attraction test that INS NPs maintained the existing magnetism. In order to further determine the impact of the wrapping process on the spectral characteristic of IR-780, the absorption and emission spectra were measured. The results are shown in Fig. 2j and Fig.S2. From the figure, it can be seen that the absorption spectrum of encapsulated IR-780 shows a slight red shift. The free IR-780 exhibits an absorption peak at 780 nm, while after encapsulation, it has its maximum absorption peak at 790 nm. This shift may be due to hydrophobic interactions or polarity changes between the near-infrared dye and the aromatic groups abundant in the PDA. This observation is consistent with the results of other researchers [39, 40]. The slight red shift will not interfere with subsequent treatment and imaging. The T2 relaxation rate of INS NPs was approximately 58.7 mM^−1^ s^−1^, indicating that the INS NPs can be applied in MRI T2-weighted imaging (Fig. 2k).

**Fig. 3:**
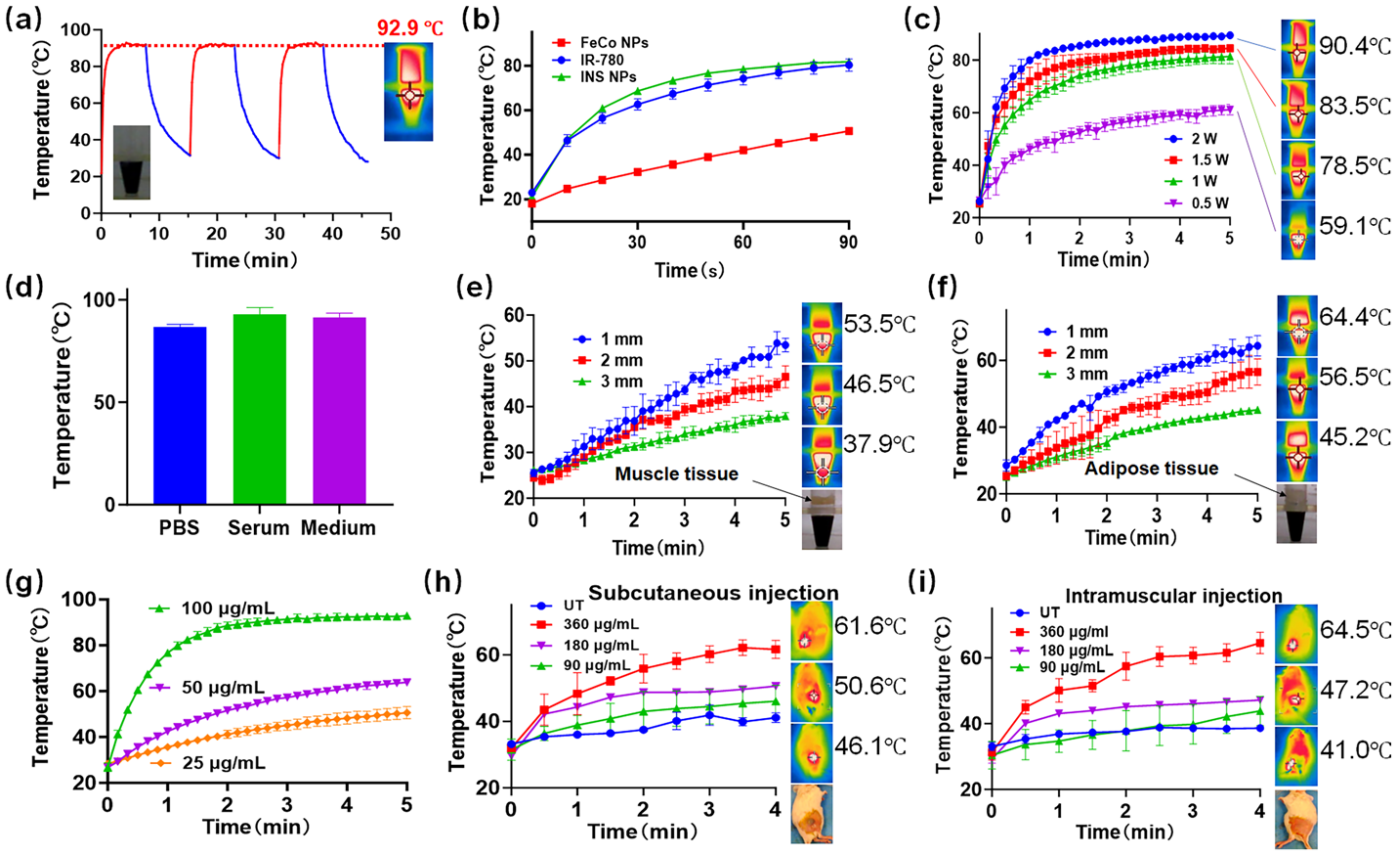
The photothermal conversion results of INS NPs (all tests are *n* = 3): **a** the thermal stability and maximum temperature rise test results of INS NPs; **b** the temperature rise curves of INS NPs and their main components; **c** the temperature rise curve of INS NPs under different irradiation powers; **d** INS NPs. The maximum temperature rise test results in PBS, serum and medium, respectively; **e** and **f** the photothermal heating curve of INS NPs under irradiation with the block of muscle and fat tissue in different thicknesses; **g** the temperature rise curves of INS NPs with different concentrations under a laser irradiation with the same power; **h** and **i** the photothermal heating curve of INS NPs at subcutaneous and intramuscular positions in mice, the sample injection volume was 100 μL. In addition to **c**, the laser power for all other groups was set at 1 W/cm^2^

The photothermal conversion performance of INS NPs was comprehensively tested. Through the photothermal stability test (Fig. 3a), we found that the maximum heating temperature of INS NPs exceeded 92 °C, and the heating was stable. There was no obvious decrease in the maximum temperature in the three consecutive excitations. Figure 3b shows a test of the photothermal conversion effects of different components of INS NPs. The results show that IR-780 was the main heat-generating substance, and FeCo NPs also have an obvious heat-generating capability. As shown in Fig. 3c and f, the photothermal conversion effect of INS NPs had a positive correlation between the concentration and power. INS NPs could also maintain very good photothermal effects in different dispersion solvents, ensuring the needs of cell and animal experiments (Fig. 3d). Since INS NPs was designed for in vivo PTT, a blocking heating experiment was conducted. From Fig. 3e and f, the results showed that INS NPs could effectively generate heat regardless of whether they were blocked by adipose or muscle tissue. The effect of muscle blocking was greater than that of adipose tissue. We observed significant heating effect after subcutaneous and intramuscular injections of INS NPs to mice (Fig. 3h and i). The temperature met the treatment need of hyperthermia.

### In vitro anti-tumor effect of INS NPs

**Fig. 4:**
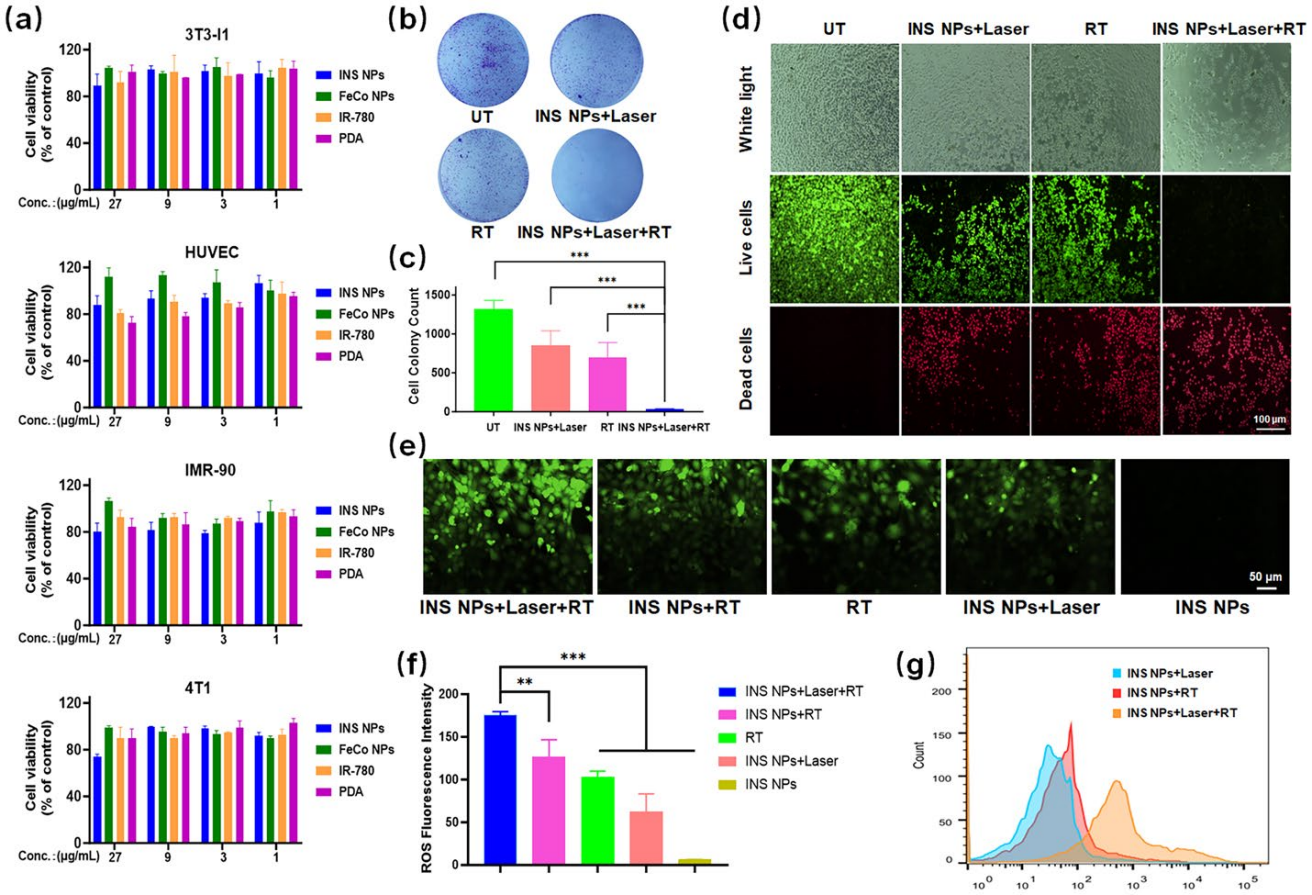
The synergistic intervention effect of INS NPs at the cellular level: **a** the cytotoxicity of INS NPs to the cell lines of 3T3-l1, HUVEC, IMR-90, and 4T1; **b** and **c** the colony formation test results of INS NPs combined with different interventions; **d** fluorescence microscope pictures of the live and dead cell staining results of INS NPs combined intervention; **e** and **f** fluorescence microscope pictures and fluorescence quantification results of intracellular ROS generation in the group of INS NPs combined with different interventions; **g** the flow cytometry results of intracellular ROS generation in the groups of INS NPs combined with different interventions (*n* = 3). For the statistically significant differences, *p* < 0.01 is marked as **, and *p* < 0.001 is marked as ***

The basic cytotoxicity of INS NPs was tested by CCK-8 assay on 5 normal cell lines (3T3-L1, HUVEC, IMR-90, MCF-10a, and HL-7702) and a TNBC cell line (4T1). The results in Figs. 4a and S3 show that INS NPs did not have obvious cytotoxicity and could be safely used for subsequent tests. The synergistic effect of INS NPs at the cellular level was then tested by colony formation and live–dead cell staining tests on 4T1 cells. The results of colony formation are shown in Fig. 4b and c. It can be seen that in the Untreatment (UT) group, when INS NPs were added alone without irradiation, a large number of cell colonies were formed after a certain period of time. After adding 808 nm laser irradiation, the highest intervention temperature during the experiment was approximately 43 °C, and the number of clones was reduced by one-third. After the cells were intervened with ionizing radiation in RT, the number of cell clones was further reduced. In the INS NPs + laser + RT group, the number of colonies was greatly reduced, resulting in a very obvious statistical difference from the three above-mentioned groups. The staining results of live and dead cells further confirmed that INS NPs had a very good synergistic intervention effect. From Fig. 4d, it can be seen that the cells in the UT group basically maintained a good survival rate, and there was obvious cell death in both the laser irradiation and RT groups, respectively. In contrast, the cell survival rate in the combined intervention group was extremely low. In order to prove the mechanism of the synergistic effect of INS NPs, an ROS indicator was applied because both RT and PDT rely on ROS. Figure 4e is a fluorescence micrograph of ROS produced in cells in different intervention groups. It can be clearly seen that the combined intervention group’s fluorescence intensity was significantly higher than those of other groups. From the quantitative analysis of fluorescence intensity, we found that there were significant differences between combined intervention group and the other four groups (Fig. 4f). In order to be quantitatively accurate, we conducted a flow cytometric analysis of the ROS levels produced in cells by INS NPs combined with different intervention methods. As shown in Fig. 4g, the intracellular ROS level in the combined intervention group was significantly higher than that of INS NPs combined with phototherapy or RT Group. These results all prove that INS NPs could realize the combined intervention of ionizing radiation RT and phototherapy and achieve a significant synergistic effect.

### Cell delivery of INS NPs

**Fig. 5:**
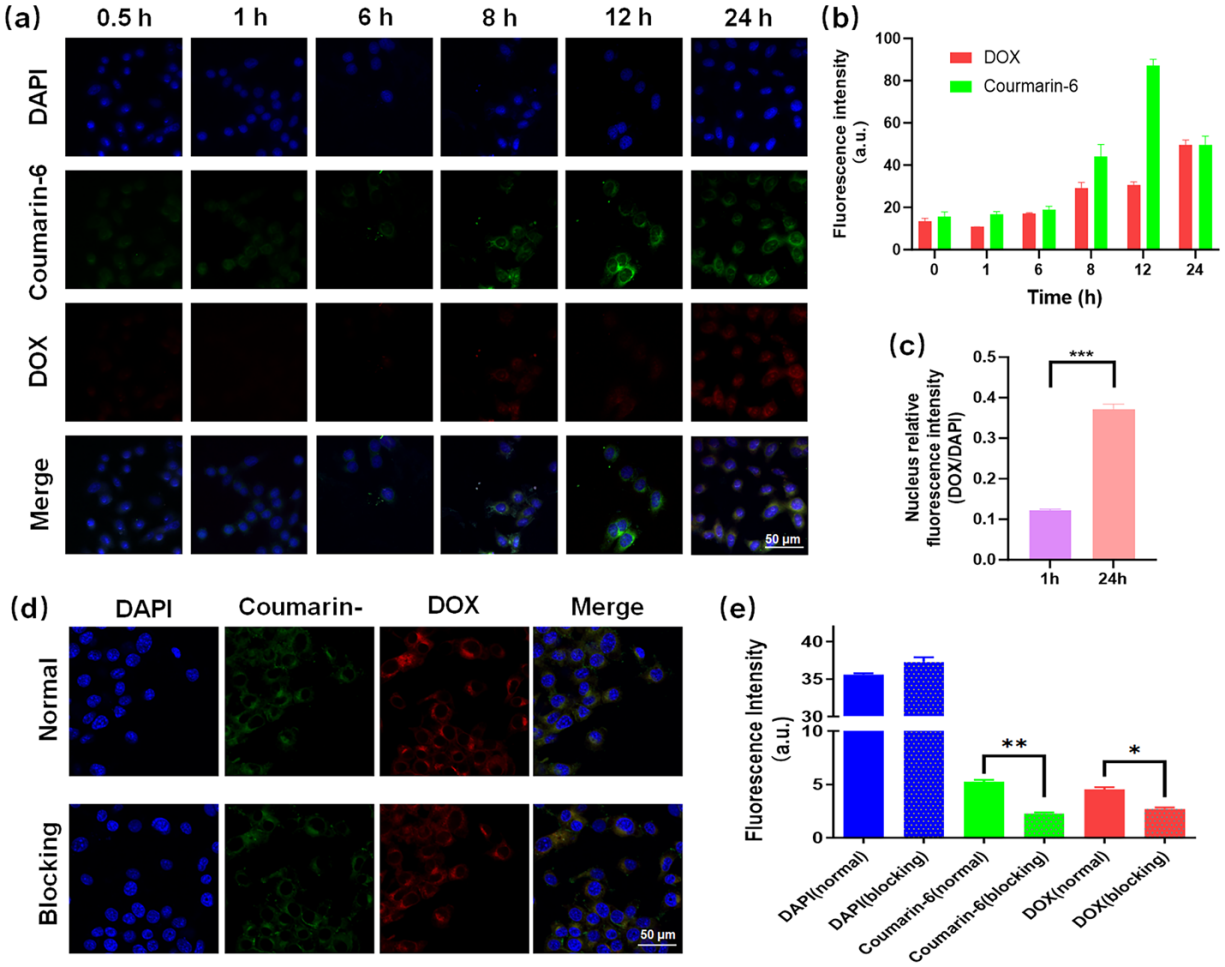
Cellular internalization and affinity test results of INS NPs. **a** The fluorescence signal accumulation after INS NPs were endocytosed by 4T1 cells; **b** the quantitative results of two fluorescence signals in cells (*n = 3*); **c** the comparison of the relative fluorescence intensity of DOX and DAPI in the nucleus at the beginning and end of the experiment (*n* = 3); **d** the fluorescence pictures of INS NPs internalization in 4T1 cells which were treated with NaN_3_, an endocytosis inhibitor; **e** the comparison of the three fluorescence signal intensities between the sodium azide-treated group and the control group (*n* = 3). For the statistically significant differences, *p* < 0.05 is marked as *, *p* < 0.01 is marked as **, and *p* < 0.001 is marked as ***

Two fluorescent molecules, coumarin-6 and DOX, were employed to label INS NPs. We designed two experiments, internalization and affinity testing, to prove that INS NPs can effectively deliver to tumor cells. The internalization results are shown in Fig. 5a. The fluorescence micrographs exhibited that the blue fluorescence signal of DAPI labeling the nucleus in each group of cells did not change throughout the experiment. In contrast, the green and red fluorescence signals in the cells increased over time. After further quantification of the fluorescence intensity (Fig. 5b), it can be seen that the overall intracellular fluorescence signal and coumarin-6 signals reached their peaks at 12 h, while the maximum DOX signal intensity was at 24 h. This phenomenon may be related to the properties of the two fluorescent molecules. Coumarin-6 is a hydrophobic molecule, which mainly bound to membrane structures in a cell and will be gradually excreted after saturation. DOX is a chemotherapeutic drug that mainly binds to DNA and is not easily excreted after binding. Therefore, we further analyzed the relative fluorescence signal (DOX/DAPI) on the cell nucleus and found that the DOX signal on the cell nucleus was clearly enhanced after 24 h (Fig. 5c), indicating that DOX was gradually released from INS NPs to the cytoplasm and entered the nucleus. To prove that the fluorescent molecules were delivered into cells by INS NPs through active transport, we used sodium azide, an inhibitor to pretreat the cells, and then added INS NPs for co-incubation. After adding the inhibitor, the cellular fluorescence signal dropped significantly. The microscopic image and fluorescence intensity quantitative analysis results are shown in Fig. 5d and e, respectively.

### Biocompatibility test of INS NPs

**Fig. 6:**
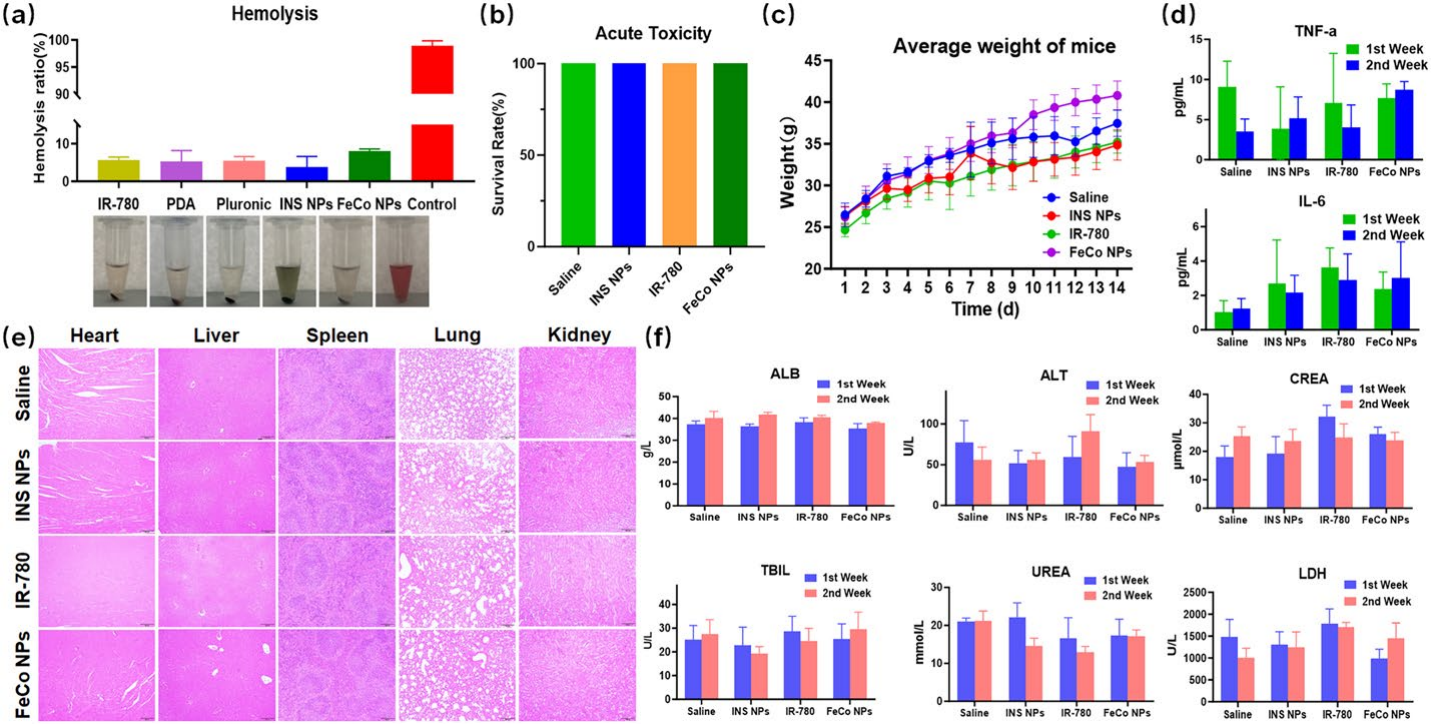
Biocompatibility test results of INS NPs. **a** The hemolytic test of INS NPs and its main components (*n* = 3); **b** the survival rate of mice in the acute toxicity test of INS NPs; **c** the average body weight change curve of mice in the acute toxicity test of INS NPs; **d** the concentrations of inflammatory factors TNF-α and IL-6 in the blood of mice during the first and second weeks of the acute toxicity test (*n = 3*); **e** the HE staining results of the main organ tissues of mice in the first week of the acute toxicity test; **f** the main blood biochemical indicators of mice in each group in the first and second weeks of the acute toxicity test (*n* = 3)

As administered intravenously, INS NPs should not cause any significant damage to red blood cells. As shown in Fig. 6a, Triton X-100 solution caused the rupture of almost all red blood cells in the positive control group, while the hemolysis rates of INS NPs and its main components were below the safety line. The hemolysis rate of INS NPs was the lowest of all the groups. The acute toxicity of INS NPs was then tested on BALB/c mice. The injection dose was 80 mg/kg. Within 14 days, the mice remained alive without obvious toxic reactions, and their body weight continued to grow steadily. The results are shown in Fig. 6b and c. We then evaluated whether INS NPs would cause pathological reactions in the major organs of mice. In terms of immunity, the levels of TNF-α and IL-6, two most representative immune factors, in the serum of mice were detected for two weeks. The results are shown in Fig. 6d. There was no significant difference of the levels of TNF-α and IL-6 of INS NPs in two weeks. In other groups, TNF-α levels fluctuated slightly, but the concentrations of both factors were at relatively low levels. The results indicated that INS NPs and their main components will not cause the body to produce immune response. Through histological observation of the main organs, we found that there were no obvious pathological changes in the main organs of all groups of mice after one week (Fig. 6e). The only difference was that there was a slight increase in lymphocytes in the livers of the INS NPs and FeCo NPs treated groups. The other pathological features such as binucleates and megakaryocytes were rare. Another histological observation was conducted two weeks later. The results are shown in Fig. S4. No obvious pathological changes were found. The blood biochemical test results are shown in Fig. 6f and Fig.S5. It can be seen that the test results of most indicators in the two weeks did not show obvious differences. Only the mice in the IR-780 group had AST and CK increased, which fell back to normal levels in the second week, indicating that there might be transient liver or heart damage and other indicators were within the normal range. In summary, INS NPs exhibited excellent biocompatibility, indicating their suitability for safe utilization in subsequent imaging and therapeutic assessments.

### In vivo distribution, imaging, and targeting delivery of INS NPs

**Fig. 7:**
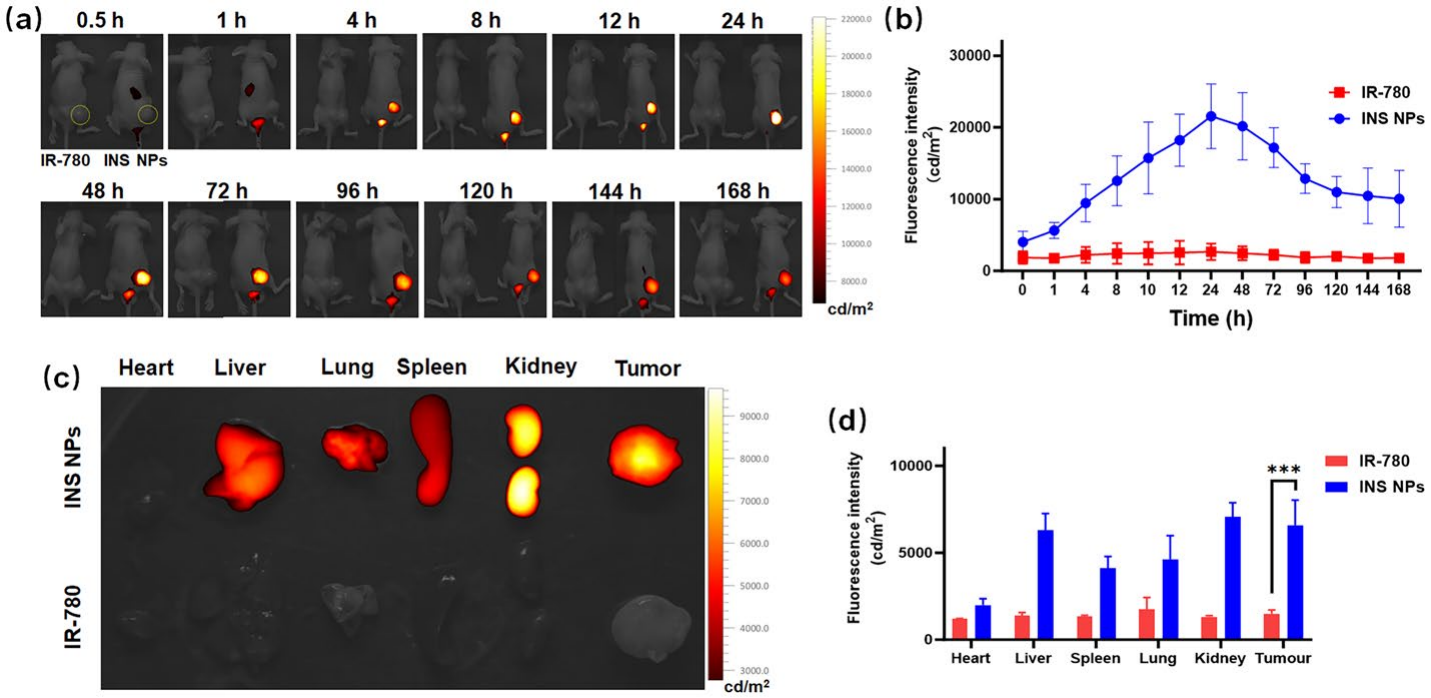
In vivo distribution and targeting delivery results of INS NPs. **a** Fluorescence signal distribution of INS NPs in mice for one week. For each time point, the left and the right mouse were injected with IR-780 and INS NPs, respectively. The yellow curve marks the tumor area. **b** The quantitative analysis of fluorescence signals in tumor area (*n* = 3); **c** the distribution of retained fluorescence signals in the major organs and tumor of mice; **d** the quantitative analysis of fluorescence signal intensity of the major organs and tumor of mice (*n* = 3). For the statistically significant differences, *p* < 0.001 is marked as ***

Single-tumor mice were selected and randomly divided into two groups. INS NPs and IR-780 were injected intravenously. The concentration of the NPs was 80 μg/mL, the injection volume was 200 μL. The concentrations of IR-780 in both groups were the same. The observation results are shown in Fig. 7a. The mice on the left and right were injected with IR-780 and INS NPs, respectively. The tumor was outlined by the yellow dotted line. It can be seen that under the same conditions, the fluorescence signal of the IR-780 group is significantly weaker than that of the INS NPs group. However, the fluorescence signal of the INS NPs group was mainly concentrated in the spleen and liver in the first 4 h. After then, the fluorescence signal started accumulating gradually in the tumor area, rose rapidly, and reached the highest peak at 24 h. The strong fluorescence signal continued to maintain until 72 h, and then began to decrease steadily. The signal lasted for more than 168 h. These results fully proved that INS NPs can not only achieve long-term circulation in the body, but can also be targeted and delivered to the tumor area. The fluorescence quantitative analysis of the tumor area is shown in Fig. 7b. It can be seen that the peak was at 24 h, and the strong signal period is maintained from 12 to 72 h. Except for the time point right after injection, the signals at other time points were significantly stronger than those in the IR-780 group. There was always a signal in the bladder area of the mice in the INS NPs group during the entire experiment, indicating the long-circulation effect of INS NPs in the body. The NPs gradually degraded and released IR-780, which was excreted through urine. Finally, the mice were euthanized and the main organs and tumor were collected. As shown in Fig. 7c, the signal of the INS NPs group was generally higher than that of the IR-780 group. The fluorescence signal in the tumor was still very strong for more than a week. In addition, the signal in the kidney was also very strong. This was consistent with the strong signal phenomenon in the bladder area during the previous in vivo imaging, demonstrating that INS NPs can achieve long circulation and slow release in vivo.

**Fig. 8:**
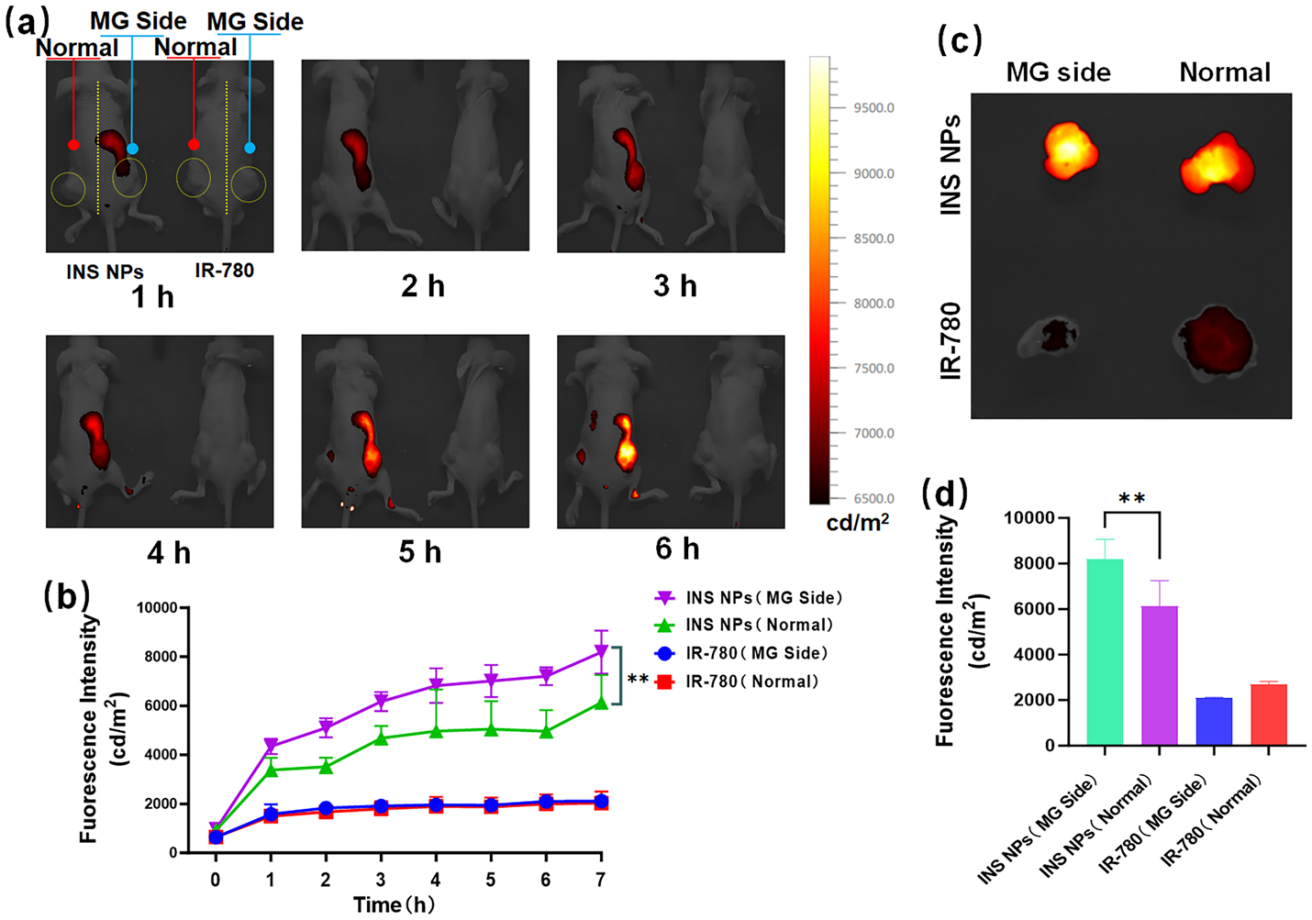
The magnetic-guided tumor targeting delivery of INS NPs. **a** The fluorescence signal distribution of INS NPs in mice after the magnetic guidance. The left and the right mouse were injected with INS NPs and IR-780, respectively. A permanent magnet was placed in the right tumor area of each mouse for magnetic guidance (Blue mark), and the left side of each mouse is non-guided (red mark). The yellow curve marks the tumor area. **b** The quantitative analysis results of fluorescence signal in the tumor area of mice (*n* = 3); **c** the quantitative analysis results of retained fluorescence signal in the tumor area of mice; **d** the quantitative analysis results of fluorescence signal in the tumor tissue (*n* = 3). For the statistically significant differences, *p* < 0.01 is marked as **

The magnetic guided targeting delivery of INS NPs was then tested. This time, mice with tumors on both sides of the crotch were selected and divided into the INS NPs group and the IR-780 group. After injection, permanent magnets were placed near the right crotch to provide guidance. As shown in Fig. 8a, with the time went by, the signal in the right tumor area gradually increased and was also significantly higher than that in the left non-magnetic guidance tumor area. The results of quantitative fluorescence signal analysis are shown in Fig. 8b. It can be seen that the tumor signals on both sides of the INS NPs group were higher than those of IR-780. This scenario was consistent with the in vivo distribution test results. In addition, the fluorescence signal intensity of the tumor area on the guiding side was significantly higher than that on the non-guiding side. Afterwards, the mice were euthanized, and the tumors were taken out for fluorescence photography and quantitative analysis of fluorescence intensity. The results are shown in Fig. 8c and d. The fluorescence signal in the tumor on the magnetic guidance side of the INS NPs group was significantly higher than that on the non-guided side, while the fluorescence signals of the two tumors in IR-780-treated mouse had no significant difference, and the signal intensity was higher in tumors on the non-guiding side. These results fully demonstrated that INS NPs have excellent magnetically guided delivery capabilities.

### In vivo anti-tumor effect of INS NPs

**Fig. 9:**
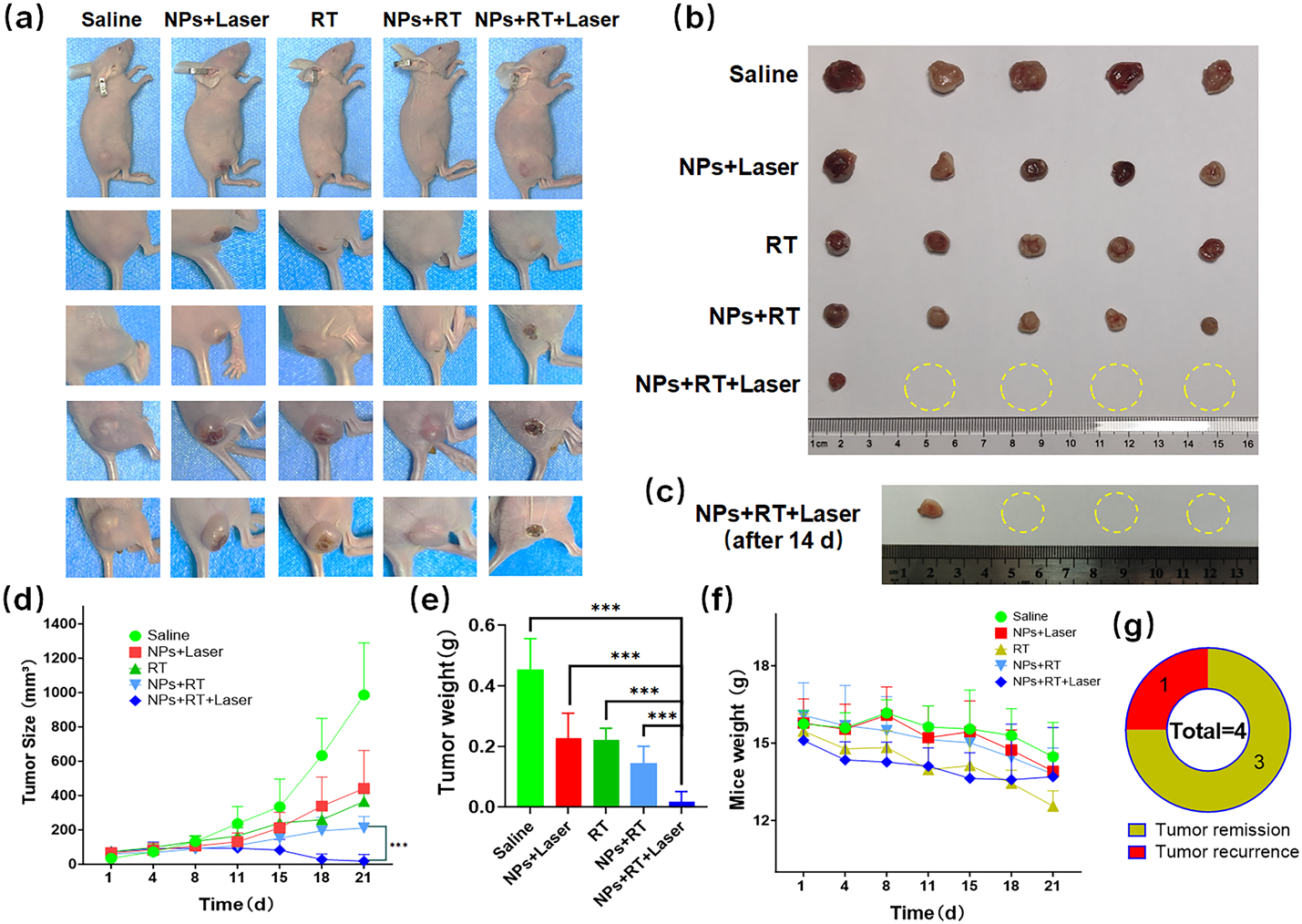
In vivo anti-tumor test results of INS NPs. **a** The photos of the tumor of mice in each group; **b** the photos of the visible tumor residue in each group of mice after the intervention; **c **the photos of the recurrent tumors of mice with invisible tumors in the combined treatment group two weeks post-intervention; **d** the growth curve of the average tumor volume of mice in each group during the intervention process; **e** the analysis results of the average weight of visible tumor residues in each group of mice after the intervention; **f** the average weight change curve of mice in each group during the intervention process; **g** the statistics on the recurrence of mice with invisible tumors in the combined treatment group two weeks post-intervention. For the statistically significant differences, *p* < 0.001 is marked as ***

The tumor-bearing mice were divided into 5 groups. The average tumor volume in each group was approximately 50 mm^3^. Different intervention methods were given to the mice. The delivery method of INS NPs was intravenous injection. The dose was 10 mg/kg, and the injection volume was 200 μL. The 808 nm laser irradiation was conducted within 18–24 h after injection with a power of 1 W/cm^2^. Magnetic guidance was performed for 4 h prior to irradiation to ensure more effective accumulation of INS NPs in the tumor region. The minimum temperature of the tumor area during excitation was 42 °C. In the combined intervention group, RT was performed 6 h after laser irradiation, and sample injection and laser irradiation were performed twice a week. During this period, the mice were photographed (Fig. 9a), and the tumor growth curve was drawn (Fig. 9d). From the results, it can be seen that the tumor volume in the saline group experienced an accelerated growth, while the tumor inhibitory effect was found in all other groups. There were also differences between the three positive control groups. In terms of the inhibitory effect, INS NPs + laser < RT < INS NPs + RT. The most important thing was that the combined intervention group had a very good therapeutic effect on tumors. 4 out of 5 mice did not have visible tumors. After calculation, we found that the average tumor volume was very small and significantly different compared with those of the other four groups. Afterwards, the mice were euthanized and the tumor were collected and weighed. The results are shown in Fig. 9b and e. It can be seen that except for the combined intervention group, the other four groups all had remaining tumors with various weights. There was only one visible tumor mass in the combined intervention group, which was the lightest among all tumors. After that, the remaining four mice in the combined treatment group were continued to be raised. After two weeks, the recurrence rate was 25% with only one mouse showed tumor recurrence (Fig. 9c and g). In addition, the average weight of mice in each group was monitored during the intervention. We found that the mice in the combined intervention group had the smallest weight loss (Fig. 9f), and even gained weight in the later period, further proving the intervention effect and in vivo safety of INS NPs.

**Fig. 10:**
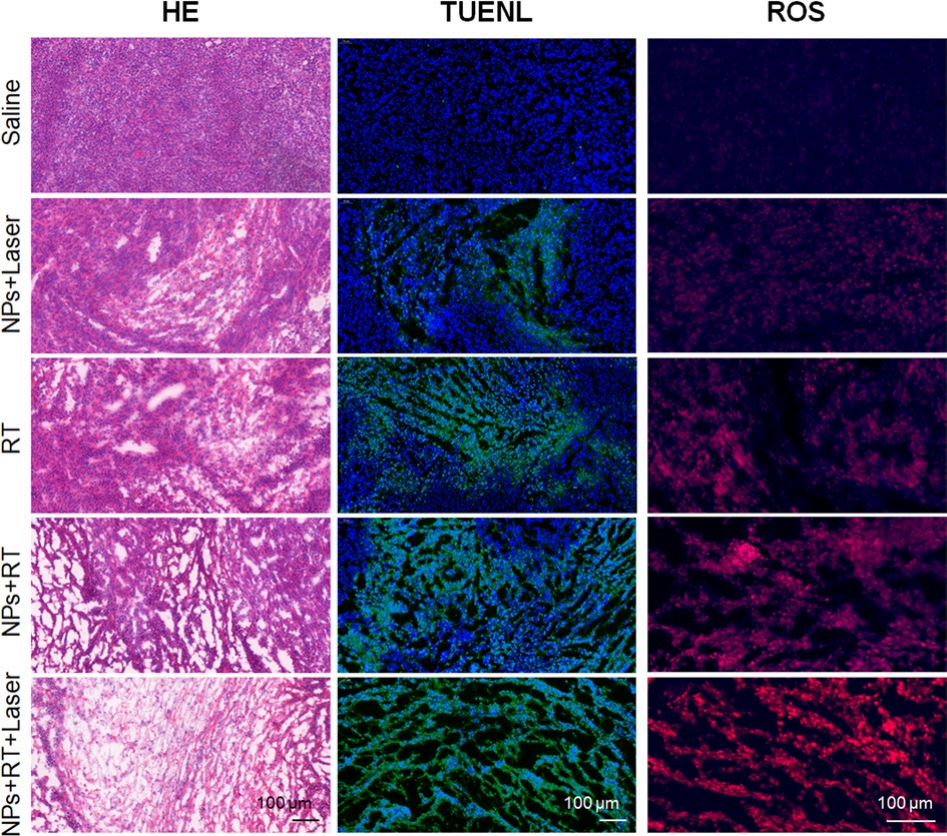
The pathological analysis results of tumor tissue in each group

Finally, the tumor tissues in each group were pathologically examined. HE staining, TUNEL staining, and ROS staining were performed, respectively. The results are shown in Fig. 10. It can be seen from the HE and TUNEL staining results that the tumor cells in the saline group were densely distributed with no obvious damage or apoptosis. After INS NPs and laser irradiation combined treatment, the PTT and PDT effects showed obvious damage to the tumor tissue, and apoptosis were observed. As a commonly used clinical treatment for tumors, RT has obvious therapeutic effects. Although the radiation dose was only 4 Gy, the damage within the tumor was significant and the degree of apoptosis increased. INS NPs can enhance the effect of RT. When RT with the same dose of 4 Gy was given, the introduction of INS NPs greatly damaged the tumor tissue and significantly enhanced apoptosis. Overall, the best effect was in the combined intervention group. In both HE or TUNEL staining results, the condition of the tumor tissue was poor, with severe tissue structure damage and a large number of cell apoptosis, fully reflecting the effect of combined treatment. ROS was used as an indicator because the both RT and PDT can cause the generation of ROS in tumors. It can be seen from the results that there were differences in ROS levels within tumors in different intervention groups. In terms of ROS production: INS NPs + laser group < RT group < INS NPs + RT group < combined intervention group. In the combined intervention group, the blue fluorescence of DHE was almost invisible, and only the red fluorescence of the oxidation product ethidium embedded in the DNA was observed. This result was highly consistent with the trend of the above-mentioned cell-level ROS test results, further proving that INS NPs have a very good synergistic intervention effect.

## Discussion

TNBC is a subtype of breast cancer characterized by the lack of expression of the ER, PR, and HER2 expression, and not suitable for endocrine therapy and targeted therapy [41]. At present, surgery is still the first choice of TNBC treatment. However, because TNBC has a higher local recurrence and metastasis rate, many patients need other prognosis approaches [42]. The important significance of RT in the treatment of TNBC is to improve local control rate, reduce recurrence rate, improve survival rate, and assist other treatments. Therefore, RT is often included in the comprehensive treatment strategy of TNBC [43]. In clinical practice, with the advancement of RT technology, tumors can be irradiated more accurately, causing less damage to the surrounding normal tissues. However, the inherent side effects of RT are still difficult to mitigate. Generally speaking, achieving good therapeutic effect with low radiation dose is the essence of RT improvement, which involves RT sensitization and enhancement. In clinical practice, chemotherapy and immunotherapy were chosen to combined with RT to enhance the therapeutic effect of RT, but there are problems with effectiveness and side effects. Therefore, it is very valuable to develop new methods that can synergize with RT. The core of RT is the use of ionizing radiation from high-energy rays for treatment. In contrast, there are also NIR methods, such as infrared, microwaves, electromagnetic waves, etc. which are also widely used in tumor treatment. Among them, the commonly used ones include PTT, PDT, and MTT [13]. Non-ionizing radiations can be used in combination to enhance the effectiveness of RT. For example, RT is often combined with thermotherapy, namely thermoradiotherapy, which increases tumor temperature, thereby increasing tumor sensitivity to RT, thus improving the efficacy of RT [44]. NIR treatment is relatively safe, but the accurate positioning is the key to maintain a good therapeutic effect. Nanoparticle drug delivery system has excellent advantages in this regard. NPs loading therapeutic agents can accumulate in tumors through targeting effect. Combining with laser, electromagnetic wave, and other irradiation methods, NPs can achieve precise intervention in the tumor area. In this study, we applied the FeCo NPs, which have good magnetic–thermal therapeutic effects, RT enhancement effect, and magnetic targeting delivery capabilities on tumors. We successfully combined FeCo NPs with RT sensitizers and photosensitizers to construct a combined treatment platform that can achieve synergistic treatment.

The FeCo NPs developed by Zhang et al. has a clear magnetocaloric effect [34]. The particle size is approximately 11 nm, which is prone to accumulate in the liver [45, 46]. The FeCo NPs have single functionality and cannot be effectively combined with other intervention methods. To this end, we modified the surface of FeCo NPs through PDA hybrid surface deposition, and loaded the photosensitizer with PTT and PDT functions into the PDA shell. Not only did we achieve a reasonable increase in particle size, but also expanded the functionality. After process optimization, the hydrodynamic size of the prepared INS NPs became 40 nm, which is very suitable for long-circulation applications in vivo. The colloidal stability was so excellent that no significant change in particle size was observed even two weeks after preparation. The in vivo distribution results confirmed the prolonged circulation effect of INS NPs. The fluorescence signal in the tumor area remained for more than a week after injection. However, once inside the cell, INS NPs can rapidly release their payload. In contrast, FeCo NPs and other iron-based nanoparticles do not have long-lasting circulation effects in vivo [47–49]. Subsequent tests also proved the advantages of INS NPs, including strong magnetism, excellent magnetocaloric effect, and magnetically guided targeting delivery. The T2 relaxation rate was 58.7 mM^−1^ s^−1^, indicating that the INS NPs have the potential for MRI T2-weighted imaging, which satisfies the multiple synergistic functions envisioned in the nanocarrier design. The encapsulation performance of INS NPs was also excellent to effectively load more IR-780 and to retain the spectral characteristics of IR-780. Multiple tests later on including photothermal conversion, photodynamic ROS generation, and fluorescence imaging have proven INS NPs’ efficacy in combination invention. In terms of photothermal conversion, the maximum temperature of INS NPs in aqueous solution was approximately 92 °C, and there was no obvious change in different solutions. Muscle and fat coverage would reduce the temperature rise, but it still met the needs of thermal therapy. Especially when blocked by fat, INS NPs had very little impact on their photothermal conversion. These results indicated that INS NPs were suitable for application in adipose tissues such as breasts. INS NPs could cause the temperature rise to an effective temperature in mice. Compared with other IR-780-loaded nanocarriers, INS NPs’ heating effect is excellent [50–52]. INS NPs are biocompatible. In vitro cell testing showed no obvious toxic reactions in all the cell lines. A similar result was found in the subsequent in vivo testing. After a large-dose injection, the mice did not show pathological reaction nor death. No pathological changes in major organs or abnormalities in inflammation levels and blood biochemical results were found in mice.

As the core functionality of INS NPs, the excellent synergy effect had received the most attention. We designed a large number of experiments to test this property and effectively proved the synergistic anti-tumor effect of INS NPs. At the cellular level, we applied lower doses for both thermal therapy and RT. The maximum temperature of thermal therapy was approximately 43 °C, which was maintained for 3 min. The dose of RT is 2 Gy. After colony formation and living and dead cell staining, we found consistent results that the combined intervention group had the most significant effect than the single treatment groups. The anti-tumor effect in vivo further accurately proved the synergistic effect of INS NPs. All RT doses in the mice tumor model were 4 Gy, and the tumor temperature after laser irradiation was approximately 42 °C, which was a low dose of intervention. Even so, the tumor volume and tumor weight of the combined intervention group were significantly different from those of the other groups. Three weeks post-intervention, 80% of the tumors in the combined intervention group achieved significant therapeutic effects in the first round of treatment. The only remaining tumor was very small. The recurrence rate of the other four mice with no visible tumor after two weeks was only 25%. Overall, 60% of the tumors in the entire group were completely eliminated. The pathological results also show that the degrees of damage and apoptosis of the remaining tumor tissue in the combined intervention group was the most severe among all groups. More importantly, compared with the gradual weight loss of mice in other groups, the weight of mice in the combined intervention group was relatively stable and even rose in the later stages. These results undoubtedly proved the excellence of INS NPs in terms of efficacy. In comparison, other reported intervention methods with the same dosage did not have such excellent results as in this study [53–55]. Furthermore, we verified the synergistic effect in terms of ROS production because both RT and PDT are related to ROS. The final results show that the amount of ROS produced by the combination treatment group was much greater than that of other positive controls, both in vitro and in vivo. The flow cytometric analysis results even showed an order of magnitude difference. The in vivo results also reflected the significant differences. These results confirmed the very good synergistic effect of INS NPs from the molecular mechanism.

## Conclusions

Aiming to solve the problems of TNBC treatment, we designed a nanoparticle that can respond to ionizing and non-ionizing radiations at the same time with combined intervention capability to achieve a synergistic treatment. Due to their good targeting and long circulation effects in vivo, INS NPs can be precisely accumulated in tumor tissues, reducing distribution and side effects of normal tissues. More importantly, INS NPs can achieve good results with low intervention doses, reducing the damage to normal tissues and improving the safety and efficiency of treatment. Overall, INS NPs, with their excellent performance, provide strong support for the development of new clinical treatments for TNBC in the future.

### Supplementary Information

Supplementary Material 1. The following supporting information can be downloaded at: www.mdpi.com/xxx/s1, Figure S1. The in vitro release results of INS NPs (n=3); Figure S2. The emission spectra of INS NPs and IR-780; Figure S3. The cytotoxicity of INS NPs on HL-7702 and MCF-10a cells (n=3); Figure S4. The HE staining results of the major organ tissues in each group of mice in the second week of acute toxicity test; Figure S5. The changes in AST and CK concentrations in each group of mice in the first and 2 weeks of the acute toxicity test (n=3). Figure S6. The original figures of in vivo distribution test.

## Data Availability

The data are contained within the article. The materials are contained within the article.

## References

[1] Schmid P, Cortes J, Pusztai L, McArthur H, Kümmel S, Bergh J, Denkert C, Park YH, Hui R, Harbeck N, Takahashi M, Foukakis T, Fasching PA, Cardoso F, Untch M, Jia L, Karantza V, Zhao J, Aktan C, Dent R, O’Shaughnessy J (2020). Pembrolizumab for early triple-negative breast cancer. N Engl J Med.

[2] Kumar P, Aggarwal R (2016). An overview of triple-negative breast cancer. Arch Gynecol Obstet.

[3] Bergin ART, Loi S (2019). Triple-negative breast cancer: recent treatment advances. F1000Res.

[4] Waks AG, Winer EP (2019). Breast cancer treatment: a review. JAMA.

[5] Bianchini G, De Angelis C, Licata L, Gianni L (2022). Treatment landscape of triple-negative breast cancer-expanded options, evolving needs. Nat Rev Clin Oncol.

[6] Pradhan R, Dey A, Taliyan R, Puri A, Kharavtekar S, Dubey SK (2023). Recent advances in targeted nanocarriers for the management of triple negative breast cancer. Pharmaceutics.

[7] Yao Y, Chu Y, Xu B, Hu Q, Song Q (2019). Radiotherapy after surgery has significant survival benefits for patients with triple-negative breast cancer. Cancer Med.

[8] Abdulkarim BS, Cuartero J, Hanson J, Deschênes J, Lesniak D, Sabri S (2011). Increased risk of locoregional recurrence for women with T1–2N0 triple-negative breast cancer treated with modified radical mastectomy without adjuvant radiation therapy compared with breast-conserving therapy. J Clin Oncol.

[9] McBride WH, Schaue D (2020). Radiation-induced tissue damage and response. J Pathol.

[10] Suwa T, Kobayashi M, Nam JM, Harada H (2021). Tumor microenvironment and radioresistance. Exp Mol Med.

[11] Xie JN, Gong LJ, Zhu S, Yong Y, Gu ZJ, Zhao YL (2018). Emerging strategies of nanomaterial-mediated tumor radiosensitization. Adv Mater.

[12] Justin CJ, Paul MH, Zachary SM (2020). The promise of combining radiation therapy with immunotherapy. Int J Radiation Oncol Biol Phys.

[13] Sowa R, Rutkowska-Talipaska J, Sulkowska U, Rutkowski K, Rutkowski R (2012). Ionizing and non-ionizing electromagnetic radiation in modern medicine. Pol Ann Med.

[14] Omer H (2021). Radiobiological effects and medical applications of non-ionizing radiation. Saudi J Biol Sci.

[15] Liang C, Xu LG, Song GS, Liu Z (2016). Emerging nanomedicine approaches fighting tumor metastasis: animal models, metastasis-targeted drug delivery, phototherapy, and immunotherapy. Chem Soc Rev.

[16] Liu JJ, Shi JJ, Nie WM, Wang SJ, Liu GH, Cai KY (2020). Recent progress in the development of multifunctional nanoplatform for precise tumor phototherapy. Adv Healthc Mater.

[17] Xie ZJ, Fan TJ, An J, Choi WS, Duo YH, Ge YQ, Zhang B, Nie GH, Xie N, Zhang TT, Chen Y, Zhang H, Kim JS (2020). Emerging combination strategies with phototherapy in cancer nanomedicine. Chem Soc Rev.

[18] Ng CW, Li JC, Pu KY (2018). Recent progresses in phototherapy-synergized cancer immunotherapy. Adv Funct Mater.

[19] Gerweck LE, Gillette EL, Dewey WC (1975). Effect of heat and radiation on synchronous Chinese hamster cells: killing and repair. Radiat Res.

[20] Overgaard J, Bichel P (1977). The influence of hypoxia and acidity on the hyperthermic response of malignant cells *in vitro*. Radiology.

[21] Horsman MR, Overgaard J (2007). Hyperthermia: a potent enhancer of radiotherapy. Clin Oncol-UK.

[22] Xiao QF, Zheng XP, Bu WB, Ge WQ, Zhang SJ, Chen F, Xing HY, Ren QG, Fan WP, Zhao KL, Hua YQ, Shi JL (2013). A core/satellite multifunctional nanotheranostic for in vivo imaging and tumor eradication by radiation/photothermal synergistic therapy. J Am Chem Soc.

[23] Zhang Z, Lo H, Zhao XY, Li WY, Wu K, Zeng FC, Li SY, Sun HZ (2023). Mild photothermal/radiation therapy potentiates ferroptosis effect for ablation of breast cancer via MRI/PA imaging guided all-in-one strategy. J Nanobiotechnol.

[24] Peng C, Liang Y, Chen Y, Qian X, Luo W, Chen S, Zhang S, Dan Q, Zhang L, Li M, Yuan M, Zhao B, Li Y (2020). Hollow mesoporous tantalum oxide based nanospheres for triple sensitization of radiotherapy. ACS Appl Mater Interfaces.

[25] Yong Y, Cheng X, Bao T, Zu M, Yan L, Yin W, Ge C, Wang D, Gu Z, Zhao Y (2015). Tungsten sulfide quantum dots as multifunctional nanotheranostics for in vivo dual-modal image-guided photothermal/radiotherapy synergistic therapy. ACS Nano.

[26] Chong LM, Tng DJH, Tan LLY, Chua MLK, Zhang Y (2021). Recent advances in radiation therapy and photodynamic therapy. Appl Phys Rev.

[27] Bulin AL, Broekgaarden M, Simeone D, Hasan T (2019). Low dose photodynamic therapy harmonizes with radiation therapy to induce beneficial effects on pancreatic heterocellular spheroids. Oncotarget.

[28] Wang Q, Liu N, Hou ZY, Shi JP, Su XH, Sun XL (2020). Radioiodinated persistent luminescence nanoplatform for radiation-induced photodynamic therapy and radiotherapy. Adv Healthc Mater.

[29] Dan Q, Hu DH, Ge YH, Zhang SY, Li SQ, Gao DY, Luo WX, Ma T, Liu X, Zheng HR, Li YJ, Shen ZH (2020). Ultrasmall theranostic nanozymes to modulate tumor hypoxia for augmenting photodynamic therapy and radiotherapy. Biomater Sci-UK.

[30] Kwatra D, Venugopal A, Anant S (2013). Nanoparticles in radiation therapy: a summary of various approaches to enhance radiosensitization in cancer. Transl Cancer Res.

[31] Khoei S, Mahdavi SR, Fakhimikabir H, Shakeri-Zadeh A, Hashemian A (2014). The role of iron oxide nanoparticles in the radiosensitization of human prostate carcinoma cell line DU145 at megavoltage radiation energies. Int J Radiat Biol.

[32] Kim DH, Nikles DE, Johnson DT, Brazel CS (2008). Heat generation of aqueously dispersed CoFe2O4 nanoparticles as heating agents for magnetically activated drug delivery and hyperthermia. J Magn Magn Mater.

[33] Fantechi E, Innocenti C, Albino M, Lottini E, Sangregorio C (2015). Influence of cobalt doping on the hyperthermic efficiency of magnetite nanoparticles. J Magn Magn Mater.

[34] Zhang LZ, Liu ZY, Liu YM, Wang Y, Tang P, Wu YS, Huang HB, Gan ZH, Liu JJ, Wu DC (2020). Ultrathin surface coated water-soluble cobalt ferrite nanoparticles with high magnetic heating efficiency and rapid in vivo clearance. Biomaterials.

[35] Lee H, Dellatore SM, Miller WM, Messersmith PB (2007). Mussel-inspired surface chemistry for multifunctional coatings. Science.

[36] Du LY, Wang XD, Liu TT, Li JY, Wang JX, Gao M, Wang HL (2019). Magnetic solid-phase extraction of organophosphorus pesticides from fruit juices using NiFe_2_O_4_@polydopamine@Mg/Al-layered double hydroxides nanocomposites as an adsorbent. Microchem J.

[37] Lin KP, Gan Y, Zhu PD, Li SS, Li C, Yu SL, Zhao S, Shi JH, Li RM, Yuan JF (2021). Hollow mesoporous polydopamine nanospheres: synthesis, biocompatibility and drug delivery. Nanotechnology.

[38] Petros R, DeSimone J (2010). Strategies in the design of nanoparticles for therapeutic applications. Nat Rev Drug Discovery.

[39] Alves CG, de Melo-Diogo D, Lima-Sousa R, Correia IJ (2020). IR780 loaded sulfobetaine methacrylate-functionalized albumin nanoparticles aimed for enhanced breast cancer phototherapy. Int J Pharmaceut.

[40] Song J, Zhang N, Zhang L, Yi HJ, Liu Y, Li YX, Li XL, Wu M, Hao L, Yang Z, Wang ZG (2019). IR780-loaded folate-targeted nanoparticles for near-infrared fluorescence image-guided surgery and photothermal therapy in ovarian cancer. Int J Nanomed.

[41] Anders C, Carey LA (2008). Understanding and treating triple-negative breast cancer. Oncology.

[42] Shekar N, Mallya P, Gowda DV, Jain V (2020). Triple-negative breast cancer: challenges and treatment options. Int J Res Pharm Sci.

[43] Yin L, Duan JJ, Bian XW, Yu SC (2020). Triple-negative breast cancer molecular subtyping and treatment progress. Breast Cancer Res.

[44] van Leeuwen CM, Crezee J, Oei AL, Franken NAP, Stalpers LJA, Bel A, Kok HP (2017). 3D radiobiological evaluation of combined radiotherapy and hyperthermia treatments. Int J Hyperther.

[45] Wang B, He X, Zhang ZY, Zhao YL, Feng WY (2013). Metabolism of nanomaterials in vivo: blood circulation and organ clearance. Accounts Chem Res.

[46] Longmire M, Choyke P, Kobayashi H (2008). Clearance properties of nano-sized particles and molecules as imaging agents: considerations and caveats. Nanomedicine.

[47] Koo SG, Park OK, Kim JH, Han SI, Yoo TY, Lee N, Kim YG, Kim HJ, Lim CH, Bae JS, Yoo J, Kim DY, Choi SH, Hyeon TH (2022). Enhanced chemodynamic therapy by Cu–Fe peroxide nanoparticles: tumor microenvironment-mediated synergistic Fenton reaction. ACS Nano.

[48] Zhang T, Jiang ZQ, Chen LB, Pan CS, Sun S, Liu C, Li ZH, Ren WZ, Wu AG, Huang PT (2020). PCN-Fe(III)-PTX nanoparticles for MRI guided high efficiency chemo-photodynamic therapy in pancreatic cancer through alleviating tumor hypoxia. Nano Res.

[49] Xie P, Huang YF, Tang KX, Wu X, Zeng C, Yang ST, Tang XH (2023). Carbon nanoparticles-Fe (II) complex for efficient theranostics of xenografted colonic tumor. Cancer Nanotechnol.

[50] Chen G, Wang KK, Zhou YW, Ding L, Ullah A, Hu Q, Sun MJ, Oupický D (2016). Oral nanostructured lipid carriers loaded with near-infrared dye for image-guided photothermal therapy. ACS Appl Mater Interfaces.

[51] Qiu XF, Xu LF, Zhang YT, Yuan AH, Wang KK, Zhao XZ, Wu JH, Guo HQ, Hu YQ (2016). Photothermal ablation of in situ renal tumor by PEG-IR780-C13 micelles and near-infrared irradiation. Mol Pharmaceutics.

[52] Hu YN, Huang SC, Zhao XJ, Chang LN, Ren XL, Mei XF, Chen ZH (2021). Preparation of photothermal responsive and ROS generative gold nanocages for cancer therapy. Chem Eng J.

[53] Luo MY, Zhu XY, Yang HF, Yan L, Cai R, Zhao YL, Tan WH (2023). Fabrication of AuPt heterostructured nanorings for enhanced synergistic radio-photothermal therapy. Nano Today.

[54] Hua SY, He J, Zhang FP, Yu JH, Zhang WX, Gao LY, Li YY, Zhou M (2021). Multistage-responsive clustered nanosystem to improve tumor accumulation and penetration for photothermal/enhanced radiation synergistic therapy. Biomaterials.

[55] Chen YJ, Meng W, Chen M, Zhang LY, Chen MW, Chen XT, Peng J, Huang NH, Zhang WH, Chen JX (2023). Biotin-decorated hollow gold nanoshells for dualmodal imaging-guided NIR-II photothermal and radiosensitizing therapy toward breast cancer. J Mater Chem B.

