# Supplementary material for: Magnetic-guided nanocarriers for ionizing/non-ionizing radiation synergistic treatment against triple-negative breast cancer
Source: Biomed Eng Online. 2024 Jul 13;23:67. doi: 10.1186/s12938-024-01263-7 (PMC11245775; doi:10.1186/s12938-024-01263-7)
Supplement: Supplementary file 1 — Supplementary Material 1. The following supporting information can be downloaded at: www.mdpi.com/xxx/s1, Figure S1. The in vitro release results of INS NPs (n=3); Figure S2. The emission spectra of INS NPs and IR-780; Figure S3. The cytotoxicity of INS NPs on HL-7702 and MCF-10a cells (n=3); Figure S4. The HE staining results of the major organ tissues in each group of mice in the second week of acute toxicity test; Figure S5. The changes in AST and CK concentrations in each group of mice in the first and 2 weeks of the acute toxicity test (n=3). Figure S6. The original figures of in vivo distribution test. [file 12938_2024_1263_MOESM1_ESM.pdf]

**Supporting information**

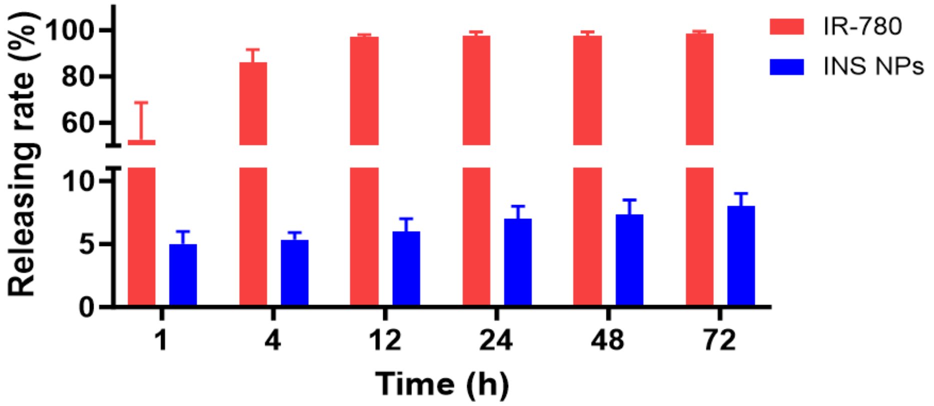


Figure S1. The *in vitro* release results of INS NPs (n=3).

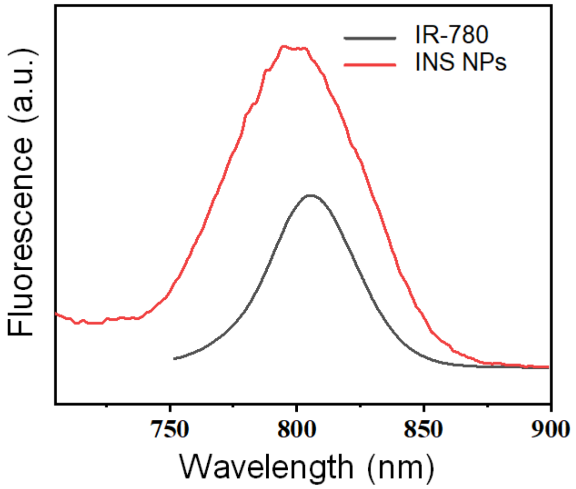


Figure S2. The emission spectra of INS NPs and IR-780.

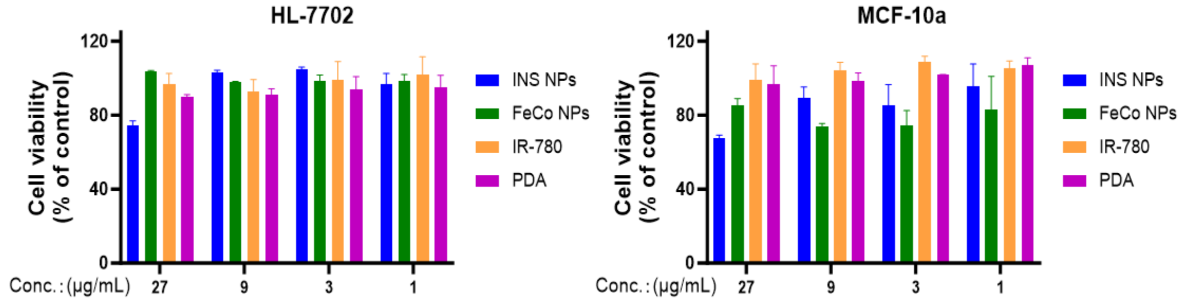


Figure S3. The cytotoxicity of INS NPs on HL-7702 and MCF-10a cells (n=3).

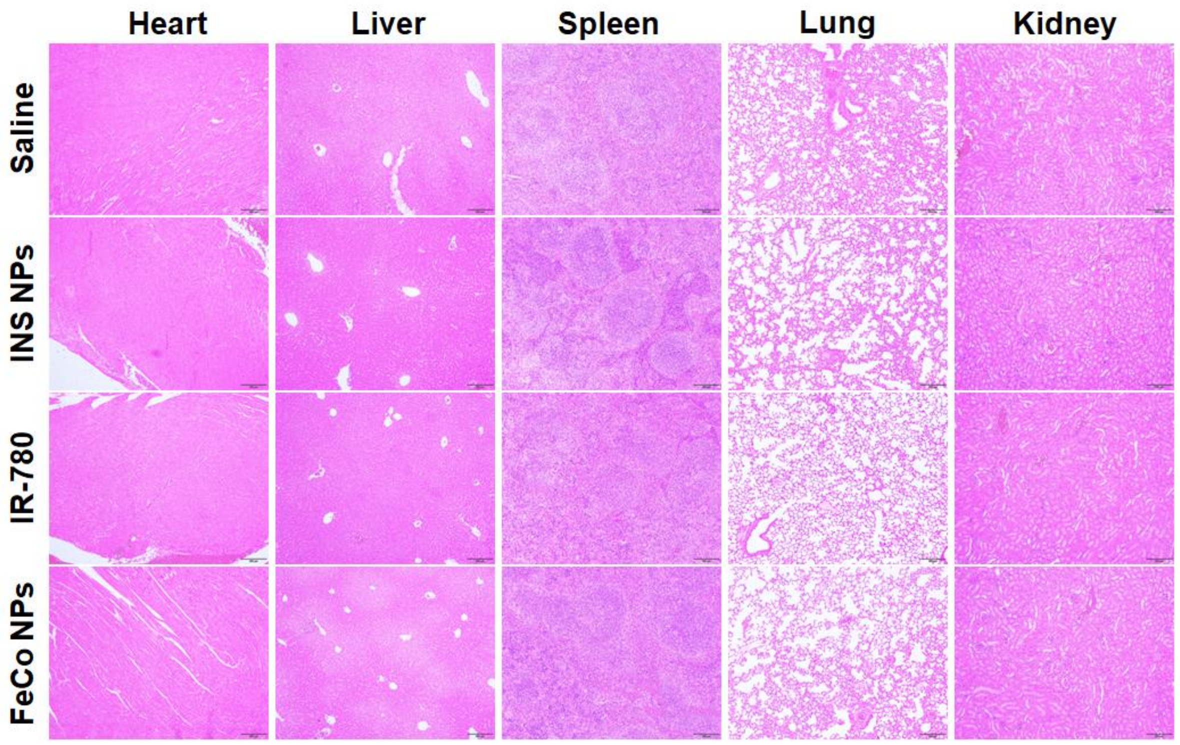


Figure S4. The HE staining results of the major organ tissues in each group of mice in the second week of acute toxicity test.

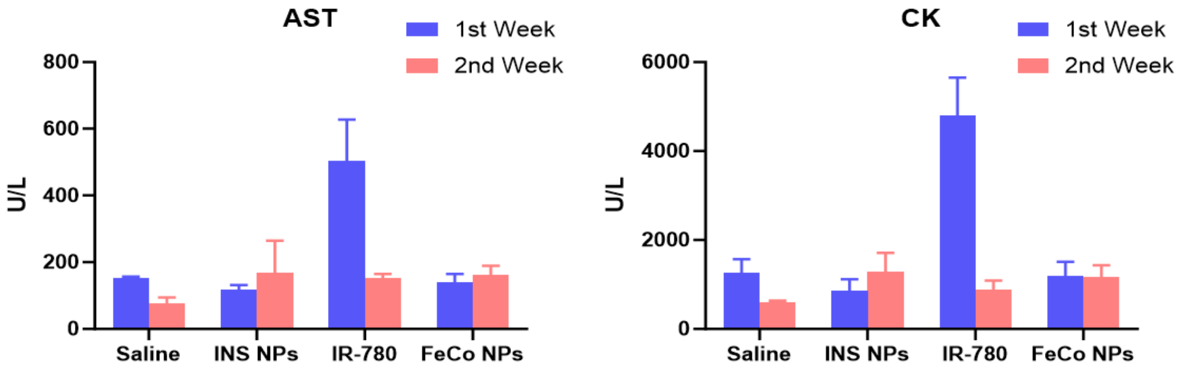


Figure S5. The changes in AST and CK concentrations in each group of mice in the first and second weeks of the acute toxicity test (n=3)

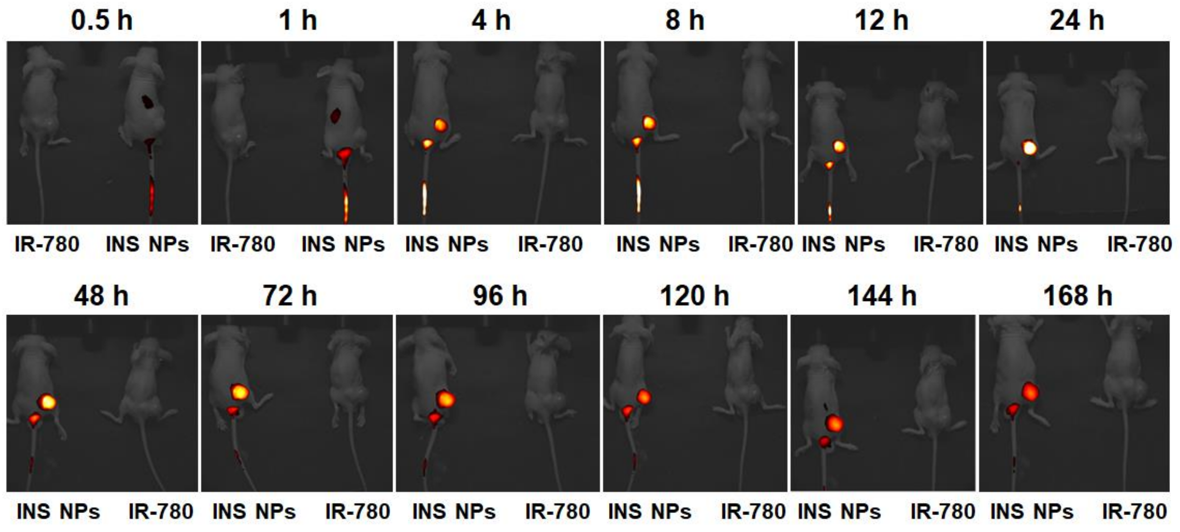


Figure S6. The original figures of *in vivo* distribution test.
